# Supplementary material for: Transmission dynamics and baseline epidemiological parameter estimates of Coronavirus disease 2019 pre-vaccination: Davao City, Philippines
Source: PLoS One. 2023 Apr 7;18(4):e0283068. doi: 10.1371/journal.pone.0283068 (PMC10081793; doi:10.1371/journal.pone.0283068)
Supplement: S1 File — This file contains solutions to the mathematical theories described in this paper. (PDF) [file pone.0283068.s001.pdf]

Transmission dynamics and baseline epidemiological parameter estimates of Coronavirus disease 2019 pre-vaccination: Davao City, Philippines

Loreniel E. Añonuevo, Zython Paul T. Lachica, Deza A. Amistas, Jayve Iay E. Lato, Hanna Lyka C. Bontilao, Jolly Mae G. Catalan, Rachel Joy F. Pasion, Annabelle P. Yumang, Alexis Erich S. Almocera, Jayrold P. Arcede, May Anne E. Mata*, Aurelio A. de los Reyes V

S1 File. Supplemental File. This file contains solutions to the mathematical theories described in this paper.

The differential equations that represent the transmission dynamics of the SARS-CoV-2 virus in the system were obtained by adding the inflow rate and subtracting the outflow rate. The following system of equations (2.1) mathematically describes the disease dynamics.

$$\left.\begin{aligned}
S'(t) &= \alpha - \delta S - \beta\left[\frac{I_H}{N} + \frac{I_M}{N}\right]S + \psi(1-r)E \\
E'(t) &= \beta\left[\frac{I_H}{N} + \frac{I_M}{N}\right]S - (\psi(1-r) + \delta_H rq + \delta_M r(1-q) + \delta)E \\
I_M'(t) &= \delta_M r(1-q)E - (\varphi_H m + \gamma_M(1-m) + \delta)I_M \\
I_H'(t) &= \delta_H rqE + \varphi_H m I_M - (\gamma_H + \mu_H + \delta)I_H \\
R'(t) &= \gamma_M(1-m)I_M + \gamma_H I_H - \delta R \\
N'(t) &= \alpha - \delta N - \mu_H I_H
\end{aligned}\right\} \quad (2.1)$$

We will show the existence of an endemic equilibrium point of the system (2.1) as follows:

Theorem 2.1. The system has an endemic equilibrium point (EE) if and only if $R_0 > 1$. Here, $EE_1 = (S^*, E^*, I_M^*, I_H^*, R^*, N^*)$ where $S^* = S_o\big(1 + b(R_o - 1)\big), E^* = \frac{\alpha(R_o-1)}{D}, I_M^* = c(R_o - 1), I_H^* = d(R_o - 1)$, $R^* = K(R_o - 1)$ and $N^* = N_0\big(1 - e(R_o - 1)\big)$.

Proof:

Let $EE_1 = (S^*, E^*, I_M^*, I_H^*, R^*, N^*)$ denote the endemic equilibrium which satisfies the following system of equations (2.2):

$$\left.\begin{aligned} \alpha-\delta S^*-\beta\left[\frac{I_H^*}{N^*}+\frac{I_M^*}{N^*}\right]S^*+\psi(1-r)E^*=0 \\ 0=\beta\left[\frac{I_H^*}{N^*}+\frac{I_M^*}{N^*}\right]S^*-[\psi(1-r)+\delta_H rq+\delta_M r(1-q)+\delta]E^*=0 \\ \delta_M r(1-q)E^*-\left[\varphi_H m+\gamma_M(1-m)+\delta\right]I_M^*=0 \\ \delta_H rqE^*+\varphi_H mI_M^*-\left(\gamma_H+\mu_H+\delta\right)I_H^*=0 \\ \gamma_M(1-m)I_M^*+\gamma_H I_H^*-\delta R^*=0 \\ \alpha-\delta N^*-\mu_H I_H^*=0 \end{aligned}\right\} \quad (2.2)$$

Solving for $S^*, E^*, I_M^*, I_H^*, R^*, N^*$, we get

$$S^*=\frac{\alpha+\psi(1-r)E^*}{\delta+\beta\left[\frac{I_H^*}{N^*}+\frac{I_M^*}{N^*}\right]} \quad (2.3)$$

$$E^*=\frac{\beta\left[\frac{I_H^*}{N^*}+\frac{I_M^*}{N^*}\right]S^*}{A} \quad (2.4)$$

$$I_M^*=\frac{\delta_M r(1-q)E^*}{B} \quad (2.5)$$

$$I_H^*=\frac{\delta_H rqE^*+\varphi_H mI_M^*}{C} \quad (2.6)$$

$$R=\frac{\gamma_M(1-m)I_M^*+\gamma_H I_H^*}{\delta} \quad (2.7)$$

$$I_M^*=\frac{\delta_M r(1-q)E^*}{B} \quad (2.8)$$

Substituting (2.5) to (2.6) yields

$$I_H^*=\frac{\delta_H rqE^*}{C}+\varphi_H m\frac{\delta_M r(1-q)E^*}{BC}=\left(\frac{\delta_H rq}{C}+\varphi_H m\frac{\delta_M r(1-q)}{BC}\right)E \quad (2.9)$$

Combining (2.3) and (2.4) yields

$$S^*=\frac{\alpha+\psi(1-r)E^*-AE^*}{\delta} \quad (2.10).$$

Substituting (2.4) with values from (2.10), (2.9), (2.8) and (2.5) gives

$$E^*=\frac{\alpha(R_o-1)}{D} \quad (2.11)$$

where $D=-\psi(1-r)R_o+AR_o-\mu_H\frac{AR_o}{\beta}+\mu_H\frac{r\delta_M(1-q)}{B}$ and $\frac{\beta(\delta_H rq+\delta_M r(1-q)+\delta)}{\mu_H A}+\frac{\beta r\delta_M(1-q)}{ABR_o}>1.$

It is now then easy to compute such that

$$I_M^*=\frac{\alpha\delta_M r(1-q)}{BD}(R_o-1),$$

$$I_H^*=\alpha\left(\frac{\delta_H rq}{CD}+\varphi_H m\frac{\delta_M r(1-q)}{BCD}\right)(R_o-1),$$

$$S^*=S_O\left(1+\frac{\psi(1-r)-A}{D}(R_o-1)\right),$$

$$R^* = \alpha\left(\frac{\delta_M r(1-q)\gamma_M(1-m)}{\delta BD} + \frac{\gamma_H \delta_H rq}{\delta CD} + \varphi_H m \frac{\gamma_H \delta_M r(1-q)}{\delta BCD}\right)(R_o - 1),$$

$$N^* = N_o\left(1 - \mu_H\left(\frac{\delta_H rq}{CD} + \varphi_H m \frac{\delta_M r(1-q)}{BCD}\right)(R_o - 1)\right).$$

Hence, the endemic equilibrium point is

$$EE_1 = (S^*, E^*, I_M^*, I_H^*, R^*, N^*)$$
$$= \left(S_o\big(1 + b(R_o - 1)\big), \frac{\alpha(R_o - 1)}{D}, c(R_o - 1), d(R_o - 1), K(R_o - 1), N_0\big(1 - e(R_o - 1)\big)\right)$$

where $b = \frac{(\psi(1-r)-A)}{D}$, $c = \frac{a\delta_M r(1-q)}{BD}$, $d = \alpha\left(\frac{\delta_H rq}{CD} + \frac{\varphi_H m \delta_M r(1-q)}{BCD}\right)$, $e = \mu_H\left(\frac{\delta_H rq}{CD} + \frac{\varphi_H m \delta_M r(1-q)}{BCD}\right)$. This completes the proof.

Stability analyses

We will prove the local stability at both equilibria of the system (2.1) as follows:

Theorem 2.2. The DFE is locally asymptotically stable if $R_0 < 1$. The endemic equilibrium point is locally asymptotically stable if $R_0 > 1$.

Proof: The Jacobian matrix of the system (2.1) is as follows:

$$J = \begin{bmatrix} -\delta - \beta\left(\frac{I_H}{N} + \frac{I_M}{N}\right) & \psi(1-r) & -\frac{\beta S}{N} & -\frac{\beta S}{N} & 0 \\ \beta\left(\frac{I_H}{N} + \frac{I_M}{N}\right) & -(\psi(1-r) + \delta_H rq + \delta_M r(1-q) + \delta) & \frac{\beta S}{N} & \frac{\beta S}{N} & 0 \\ 0 & \delta_M r(1-q) & -(\varphi_H m + \gamma_M(1-m) + \delta) & 0 & 0 \\ 0 & 0 & \delta_H rq\ \varphi_H m & -(\gamma_H + \mu_H + \delta) & 0 \\ 0 & 0 & \gamma_M(1-m) & \gamma_H & -\delta \end{bmatrix} \quad (2.13)$$

which at the DFE becomes

$$J_{DFE} = \begin{bmatrix} -\delta & \psi(1-r) & -\beta & -\beta & 0 \\ 0 & -(\psi(1-r) + \delta_H rq + \delta_M r(1-q) + \delta) & \beta & \beta & 0 \\ 0 & \delta_M r(1-q) & -(\varphi_H m + \gamma_M(1-m) + \delta) & 0 & 0 \\ 0 & \delta_H rq & \varphi_H m & -(\gamma_H + \mu_H + \delta) & 0 \\ 0 & 0 & \gamma_M(1-m) & \gamma_H & -\delta \end{bmatrix}$$

Then, the characteristic polynomial of the Jacobian matrix is solved such that $det\ (J_{DFE} - \lambda I) = 0$ where λ is the eigenvalue and I is the identity matrix. Solving and simplifying

$$\det(J_{DFE} - \lambda I) = \det\begin{bmatrix} -\delta - \lambda & \psi(1-r) & -\beta & -\beta & 0 \\ 0 & -(\psi(1-r) + \delta_H rq + \delta_M r(1-q) + \delta) - \lambda & \beta & \beta & 0 \\ 0 & \delta_M r(1-q) & -(\varphi_H m + \gamma_M(1-m) + \delta) - \lambda & 0 & 0 \\ 0 & \delta_H rq & \varphi_H m & -(\gamma_H + \mu_H + \delta) - \lambda & 0 \\ 0 & 0 & \gamma_M(1-m) & \gamma_H & -\delta - \lambda \end{bmatrix} \quad (2.14)$$

Applying Gaussian elimination and matrix operations, we can transform (2.14) into

$$\det(J_{DFE} - \lambda I) = -(-\delta - \lambda)^2(A(R_0 - 1) - \lambda)F\Omega\left(\frac{BC}{\beta(\varphi_H m + C)}\right) \quad (2.15)$$

where $F = \beta - \frac{g\lambda(A+\lambda)}{A(R_0-1)-\lambda}$ and $\Omega = \frac{\beta\delta_H rq\lambda}{C(A(R_0-1)-\lambda)} - C - \lambda - \left(\beta - \frac{\beta\lambda(A+\lambda)}{C(A(R_0-1)-\lambda)}\right)\left(\frac{\varphi_H m\lambda(A(R_0-1)-\lambda)}{\beta(A(R_0-1)-\lambda)-g\lambda(A+\lambda)} + \frac{g\delta_H rq\lambda}{\beta(A(R_0-1)-\lambda)-g\lambda(A+\lambda)}\right)$

Equating (2.15) to zero and simplifying it, we get the eigenvalues

$$\lambda_1 = -\delta < 0, \lambda_i = X_i - Y, X_i < Y, i = 2, 3, 4$$

$$\lambda_5 = \frac{A\beta(\varphi_H m + C)(R_0 - 1)}{BC},$$

which are less than zero when $R_0 < 1$. This proves that the DFE is locally asymptotically stable if $R_0 < 1$. Similarly, for the endemic equilibrium, we applied the Gaussian elimination method to the Jacobian matrix (2.13) into

$$J_{EE} = \begin{bmatrix} -\delta - \beta\left(\frac{I_H^*}{N^*} + \frac{I_M^*}{N^*}\right) & \psi(1-r) & -\frac{\beta S^*}{N^*} & -\frac{\beta S^*}{N^*} & 0 \\ \beta\left(\frac{I_H^*}{N^*} + \frac{I_M^*}{N^*}\right) & -(\psi(1-r) + \delta_H rq + \delta_M r(1-q) + \delta) & \frac{\beta S^*}{N^*} & \frac{\beta S^*}{N^*} & 0 \\ 0 & \delta_M r(1-q) & -(\varphi_H m + \gamma_M(1-m) + \delta) & 0 & 0 \\ 0 & 0 & \delta_H rq\, \varphi_H m & -(\gamma_H + \mu_H + \delta) & 0 \\ 0 & 0 & \gamma_M(1-m) & \gamma_H & -\delta \end{bmatrix}$$

then solve for the eigenvalues of $\det(J_{EE} - \lambda I)$. When simplified, we get

$$\det(J_{EE} - \lambda I) = (-\lambda - \delta)(-\lambda - 1)\left(-\lambda - \frac{C}{\varphi_H m} - 1\right)\left(-\lambda - \frac{\delta}{\gamma_M(1-m)}\right)\left(-\lambda + \frac{R_O S}{N} - 1 + \frac{\beta}{A}\left[\frac{I_H^*}{N^*} + \frac{I_M^*}{N^*}\right]\frac{\psi(1-r) - A}{\delta}\right)$$

Thus, $\det(J_{EE} - \lambda I) = 0$ has solutions $\lambda_1^* = -\delta$, $\lambda_2^* = -1$, $\lambda_3^* = -\frac{C}{\varphi_H m} - 1$, $\lambda_4^* = -\frac{\delta}{\gamma_M(1-m)}$, $\lambda_5^* = -\frac{R_O S}{N} + 1 - \frac{\beta}{A}\left[\frac{I_H^*}{N^*} + \frac{I_M^*}{N^*}\right]\frac{\psi(1-r)-A}{\delta} = -\frac{\left(1 + R_O b + e + \frac{\beta(c+d)(\psi(1-r)-A)}{AS_O\delta}\right)}{1-e(R_O-1)}(R_o - 1) < 0$ when $R_o > 1$. This holds since $1 - e(R_o - 1)$ is always nonnegative as $S^* = S_0\big(1 - e(R_o - 1)\big) \geq 0$. This completes the proof.

Note that aside from the derived closed form expressions of the reproduction numbers, disease free and endemic equilibria and its stability, these were also tested and solved using matlab symbolic computation packages and further reviewed using the Routh Hurwitz Criteria.
