# Supplementary material for: Transmission dynamics and baseline epidemiological parameter estimates of Coronavirus disease 2019 pre-vaccination: Davao City, Philippines
Source: PLoS One. 2023 Apr 7;18(4):e0283068. doi: 10.1371/journal.pone.0283068 (PMC10081793; doi:10.1371/journal.pone.0283068)
Supplement: Attachment — Submitted filename: Response to Reviewers.pdf [file pone.0283068.s003.pdf]

Transmission dynamics, baseline epidemiological parameter estimates of Coronavirus disease 2019 pre-vaccination: Davao City, Philippines

Loreniel E. Añonuevo, Zython Paul T. Lachica, Deza A. Amistas, Jayve Iay E. Lato, Hanna Lyka C. Bontilao, Jolly Mae G. Catalan, Rachel Joy F. Pasion, Annabelle P. Yumang, Alexis Erich S. Almocera, Jayrold P. Arcede, May Anne E. Mata, Aurelio A. de los Reyes V

RESPONSE TO REVIEWERS' COMMENTS

November 20, 2022

Greetings!

We would like to express our gratitude to the editors and reviewers for the beneficial suggestions. We have tried to incorporate all recommendations in the latest revision. The title of the paper herein has been revised to read "Transmission dynamics, baseline epidemiological parameter estimates of Coronavirus disease 2019 pre-vaccination: Davao City, Philippines." It was formerly "Transmission dynamics and baseline epidemiological parameter estimates of Coronavirus disease 2019 (COVID-19) pre-vaccination: Davao City, Philippines." Aside from this change, other specific responses and actions are detailed in the following sections.

REFEREE 1

We thank the reviewer for carefully reading our manuscript providing constructive suggestions for edits. We have addressed all your comments below indicating changes that we have made to the manuscript in blue.

Comments

1. Lines 107 and 116 are contradictory. How can I_H and I_M have close contact with S if they are isolated? How about considering another compartment (Q), which denotes individuals who are isolated and not infectious?

   Response: We are grateful for the keen eyes for which you've spotted this inconsistency. We apologize for the confusion the term 'isolated' has brought about. It was not correctly stated. We have deleted this term to avoid confusion and to emphasize that the compartments I_H and I_M refer to infectious individuals (whether or not they are isolated). A more elaborate descriptions of I_H and I_M in the context of Davao City are found in Page 4, Lines 101-125 in the revised manuscript. The suggested compartment Q is splendid but we do not find it necessary for our context given the limited data available to fit to the Q compartment.

   <u>Model formulation (Page 4, lines 101-125):</u>
   In this paper, the closed homogeneous population was divided into five population classes, namely, susceptible (S), quarantined/exposed (E), infectious individuals treated in either a monitoring facility (I_M) or hospitals handling severe cases (I_H), and recovered (R) individuals. We specifically defined

the compartments as follows:

Susceptible (S): Individuals who have not been infected with COVID-19 but are at risk of contracting the disease;
Exposed (E): Individuals who were under quarantine who may have been exposed to the disease and were still in the latent stage or who may have been exposed but have not necessarily contracted the virus;
Hospitalized (I_H): Infected individuals confirmed positive for COVID-19 treated in COVID-19-designated referral hospitals and exhibited moderate to critical symptoms or have known comorbidities;
Monitored (I_M): Infected individuals confirmed positive for COVID-19 under monitoring in designated Temporary Treatment and Monitoring Facilities (TTMFs) . These are infected persons who exhibited no symptoms (asymptomatic) or who had mild COVID-19 symptoms; and
Recovered (R): Individuals who recovered from the disease and possessed a certain level of immunity.

The schematic diagram in Fig **??** depicted the disease transmission dynamics, with the corresponding parameter descriptions, range of values, and sources, were detailed in Table **??**. Herein, the mean residence time in E, in I_M, and in I_H are respectively given as
$A^{-1} = (\psi(1-r) + \delta_H rq + \delta_M r(1-q) + \delta)^{-1}$,
$B^{-1} = (\varphi_H m + \gamma_M(1-m) + \delta)^{-1}$, and
$C^{-1} = (\mu_H + \delta + \gamma_H)^{-1}$.

2. One of the main contributions of the paper is introducing the parameter r. This is interesting and realistic given the situation in Davao. I recommend that the author perform more simulations on this. For example, do some scenarios for varying r and see the effects on the number of cases. This might add merit to the work.

Response: Thank you so much for your suggestion. We gladly incorporated this in the revised manuscript. Please see Page 11 Lines 364–377. One thing to note is that the parameter r primarily affects the transient dynamics of the system. This is valid since the city varies its intervention efforts every two weeks thus it is impossible for one to perceive the disease's steady state behaviour. A figure showing simulations of exposed, hospitalized infectious, and mild infectious for different values of r is presented in Figure 5.

Epidemiological parameters (Page 11 Lines 364-377):

Different scenarios were considered concerning the different r values estimated as shown in Fig 4. It shows that if mass testing was done to at least halve the positivity rate, the daily new exposed population would have reduced from 73 to 21 individuals after 60 days of Q1 and from 34 to 14 individuals during Q2; the daily new I_M cases would have reduced from 24 to 5 individuals after 60 days of Q1 and from 30 to 8 individuals during Q2; and the daily new I_H cases would have become 0 from 2 individuals after 60 days of Q1 and 0 indv from 1 individuals during Q2. Moreover, if the efforts were a bit laxer to twice than what was the positivity rate, the daily new exposed population would have increased to 358 individuals after 60 days of Q1 and to 90 individuals during Q2, the daily new I_M cases would have increased to 175 individuals after 60 days of Q1 and to 122 individuals during Q2, while the daily new I_H cases would have become 13 individuals after 60 days of Q1 and 3 individuals during Q2. These results corroborate with the need for mass testing, contact tracing, and the need for faster release of test results towards mitigating the disease [54].

3. The figures are poorly generated. Some of the legends are not readable.

Response: Thank you so much for this comment. We have readily updated our figures and tried to make all of its components, including the legends, to be distinguishable. Please see the new figures 1-6.

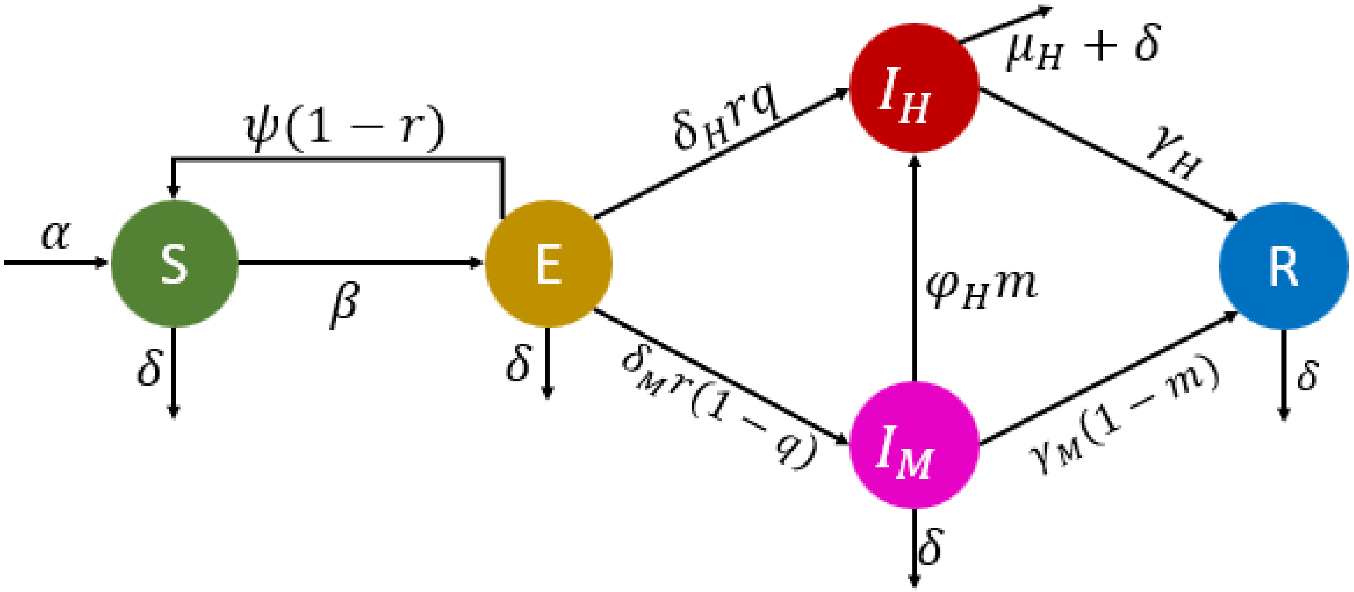

Figure 1: **Schematic diagram of the Regional COVID-19 transmission dynamics.**
Circles represent the compartments (state variables of the model), while arrows indicate flows between compartments. The outflow from I_H indicated by μ_H indicates disease-induced mortality.

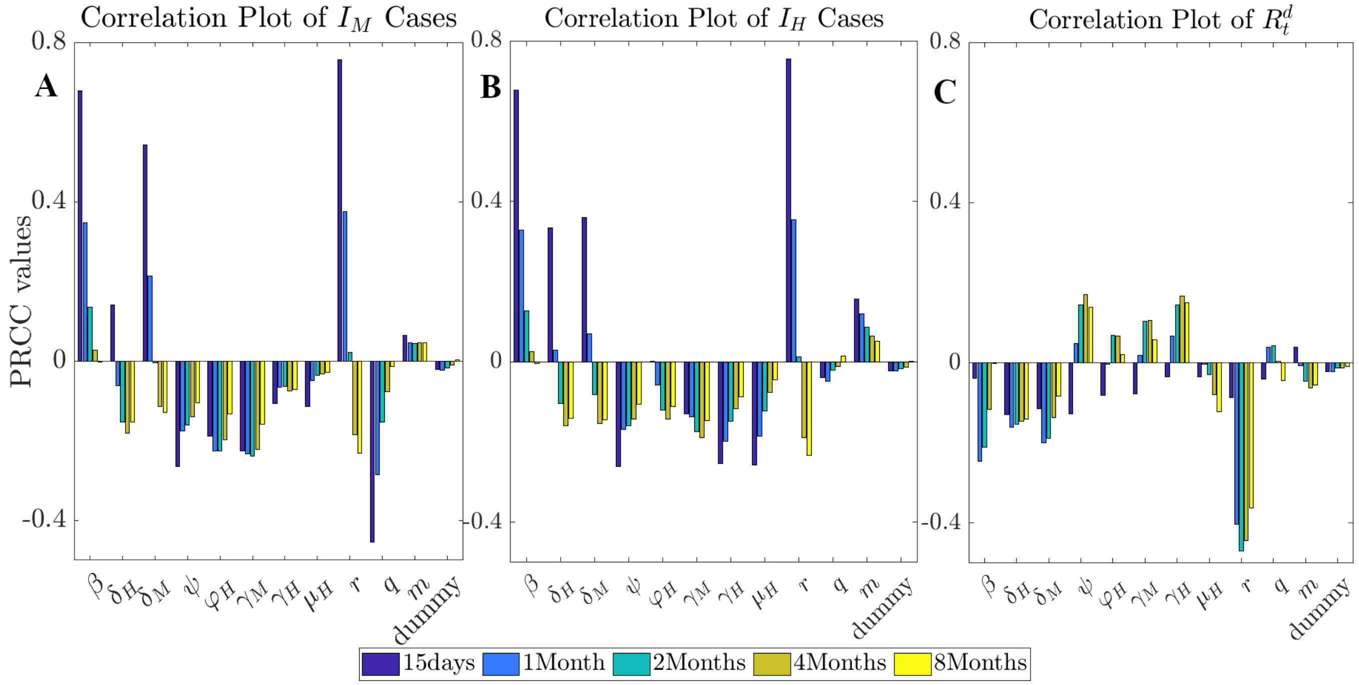

Figure 2: **The PRCC of model parameters.**
Using 10,000 LHS samples for the period March 8, 2020, to March 5, 2021, for (A) the number of monitored cases, (B) hospitalized cases, and (C) the deterministic time-varying reproduction number (R_t^d). The uncertainty and sensitivity analysis was performed via the LHS/PRCC method.

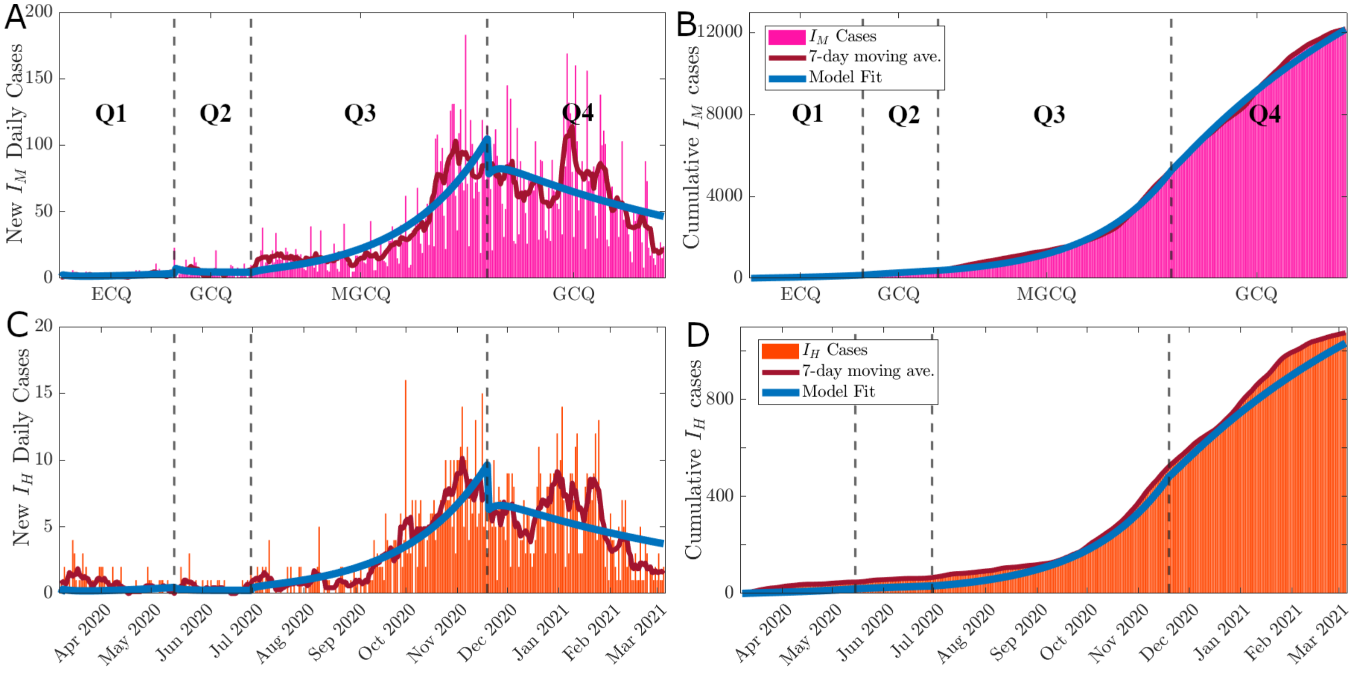

Figure 3: **The best fit of the SEI_HI_MR model to the daily new cases according to health status upon admission.**
The four quarantine periods are separated by broken lines. The bars are the daily new incidence data in Davao City, while the solid blue line corresponds to the model simulations. It shows the model fit vs daily data (A), the model fit vs. cumulative data for monitored cases (B), the model fit vs. daily data (C), and the model fit vs. cumulative data for hospitalized cases (D).

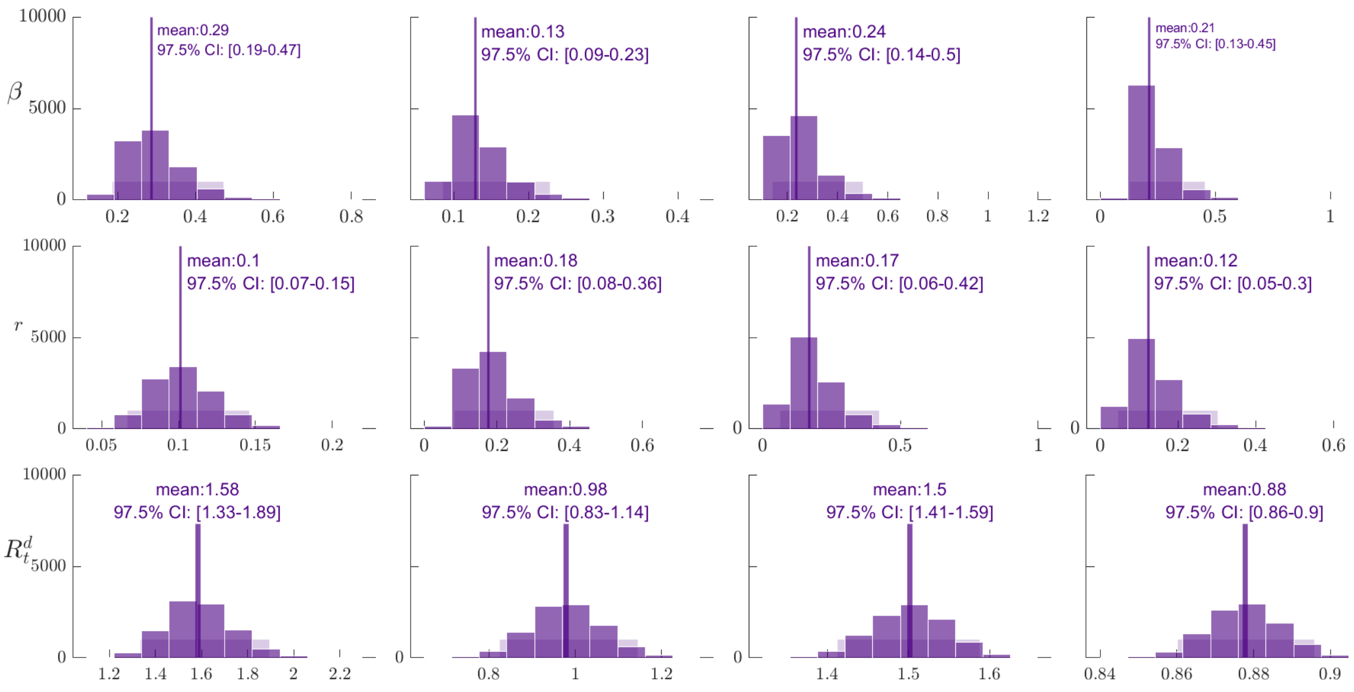

Figure 4: **The histograms obtained from the parametric bootstrapping approach.**
These show the empirical distributions of the estimated parameters β and r, including R_0 for (from up to down) Q1, Q2, Q3, and Q4 estimations, respectively. We characterized the empirical distributions using 10,000 Bootstrap realizations assuming a Poisson error structure.

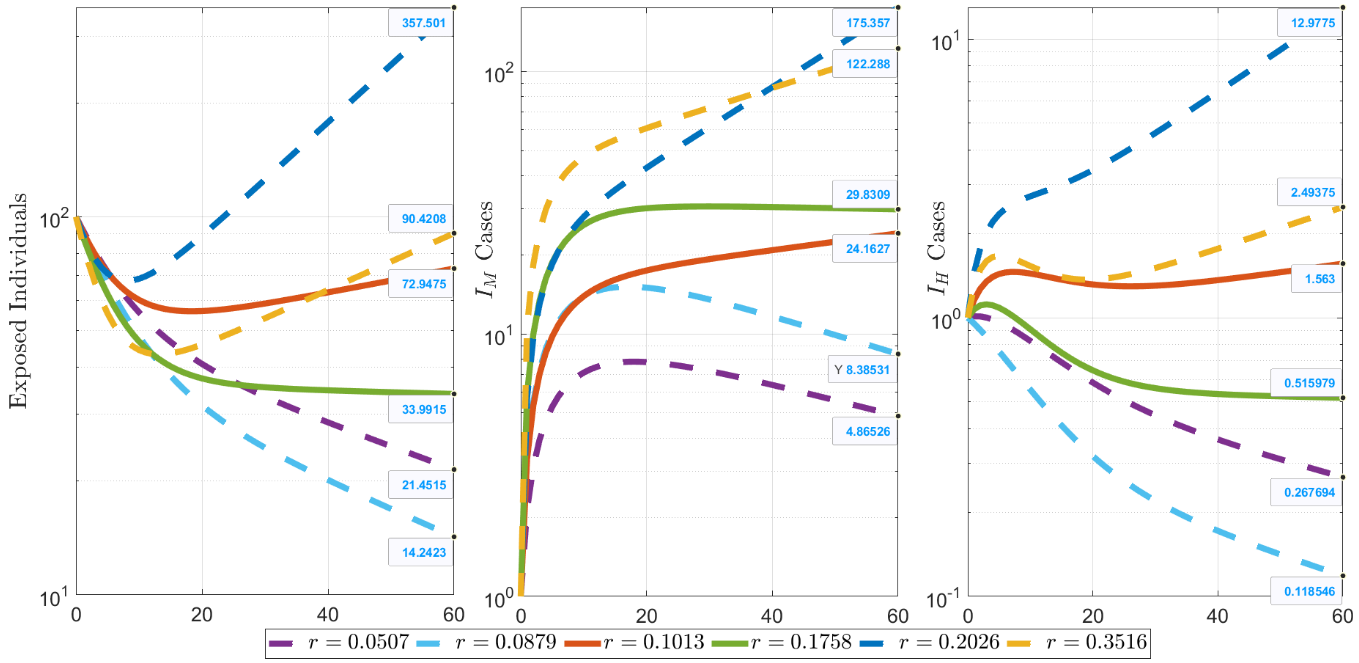

Figure 5: **The different simulations of r values and its effect on the transmission dynamics in logscale.** These show the epidemic curves using the smallest estimated $r = 0.1013$, biggest estimated $r = 0.1758$, and scenarios where the r values are half ($r = 0.0507$, $r = 0.0879$), and twice the corresponding estimates ($r = 0.2026$, $r = 0.3516$). Solid curves are the simulations using r values from the model estimates, while the dashed curves are simulations using arbitrarily chosen r values for scenario analysis.

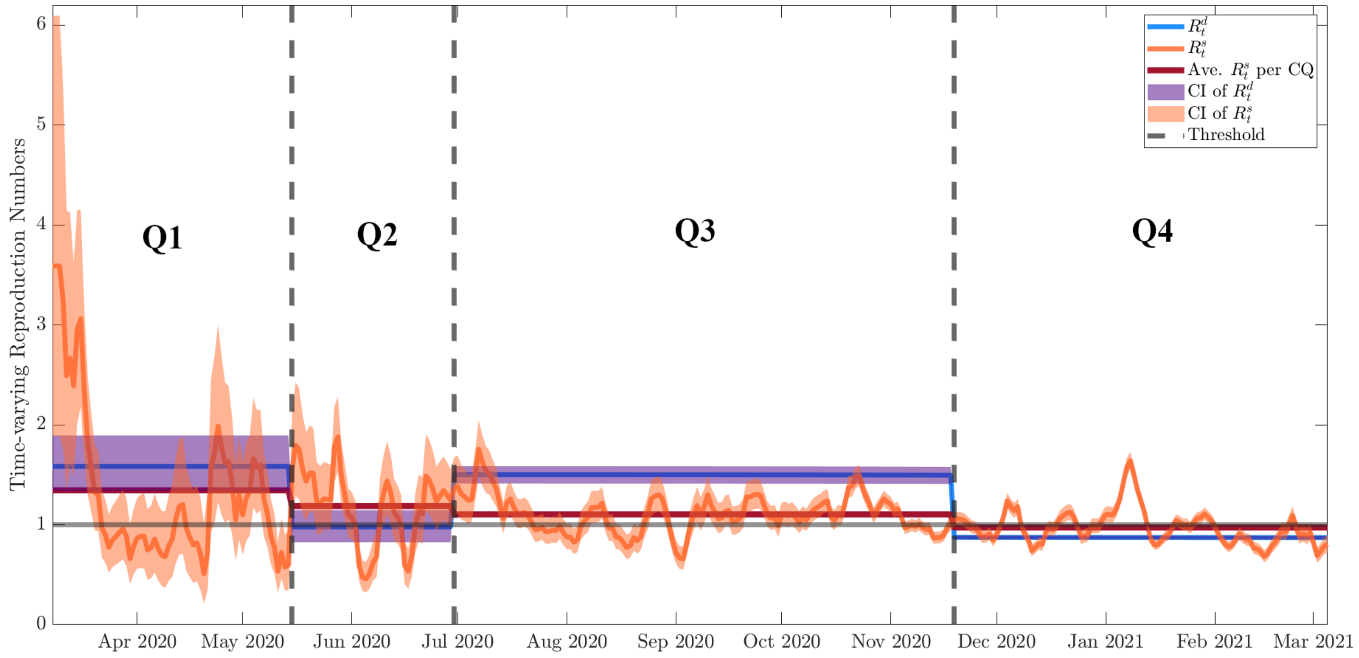

Figure 6: **The deterministic reproduction number R_t^d from the SEI_HI_MR model against the statistical reproduction number R_t^s plot for the time periods considered.**

4. Lines 210-211 and Table 1 mention that some of the parameters were statistically solved from the data and a reference [37] was cited. However, this reference is not available online and seems like a study made by the research group. I suggest that the authors add a supplementary file detailing how

each of the statistically derived parameters is calculated.

Response: Thank you for this suggestion. The observation is reasonable. The referenced material is really a guide made by the research group in calculating the parameter values. Since it is unavailable online, and along with the comment of reviewer 2, we are instead citing two papers which made a similar approach and were also references of the guide crafted. Please see Page 7 and Lines 205-207 and references [42,43].

Sensitivity analysis (Page 7, lines 205–207):

Other parameter values were either estimated from the data [42, 43] or taken from existing published literature.

5. Lines 211-212 mention that some of the parameters are obtained from existing published literature? Which parameters? Which references? The authors may add another column in Table 1 to indicate the sources of the parameters.

Response: We are grateful for this observation. We really did put in the reference in the former Table 1 but because of your comment, we realized that it wasn't presented clearly. Thank you for pointing this out. Hence, we have revised the table as shown in the new tables 2 & 3.

Table 2:(Page 5):

Table 1: **Parameter description, values, and sources for Davao City, Philippines.**

Parameter	Description	Value/Range	Unit	Source
N	Total Population	1,816,987	indv	[33]
β	Transmission rate	$[0, 10]$	per day	Estimated
r	Proportion of Exposed that becomes infectious	$[0, 1]$	% per day	Estimated
δ_H	$E \to I_H$: Reciprocal of the period from onset of illness to Hospital Admission	$[0, 1]$	per day	*
δ_M	$E \to I_M$: Reciprocal of the period from onset of illness to TTMF Admission	$\frac{1}{3}$,$[0, 1]$	per day	*
γ_H	$I_H \to R$: Recovery rate of I_H individuals	$[0, 1]$	per day	*
γ_M	$I_M \to R$: Recovery rate of I_M individuals	$[0, 1]$	per day	*
μ_H	$I_H \to D$: COVID-19 related death rate	$[0, 1]$	per day	*
q	Proportion of Reported cases that are Moderate-Critical	$[0, 1]$	% per day	*
m	Proportion of I_M cases that become Critical/Severe	$[0, 1]$	% per day	*
ψ	Discharging rate of Exposed	$\frac{1}{14}$	per day	[14]
φ_H	$I_M \to I_H$: Hospitalization rate of I_M	$\frac{1}{5}$	per day	*
δ	Natural Death Rate	$\frac{1}{26017.2}$	per day	[34]
α	Recruitment Rate	69.8379**	per day	[34]
A^{-1}	Mean residence time in E	$[0, 20]$	days	Calculated
B^{-1}	Mean residence time in I_M	$[0, 20]$	days	Calculated
C^{-1}	Mean residence time in I_H	$[0, 20]$	days	Calculated

* estimated from the data, $^{**}\alpha \approx N\delta$

Table 2: **Estimated parameter values per Quarantine Period for Davao City, Philippines.**

Parameter	Q1 (ECQ)	Q2 (GCQ)	Q3 (MGCQ)	Q4 (GCQ)
β	0.2862	0.1284	0.2359	0.2142
r	0.1013	0.1758	0.1702	0.1238
δ_H	$\frac{1}{7}$	$\frac{1}{5}$	$\frac{1}{3}$	$\frac{1}{3}$
γ_H	0.0238	0.0257	0.0244	0.0193
γ_M	$\frac{1}{19}$	$\frac{1}{16}$	$\frac{1}{14}$	$\frac{1}{11}$
μ_H	0.1223	0.1579	0.2928	0.3002
q	0.2271	0.0788	0.0842	0.0742
m	0	0	0.0002	0.0054

6. **Please comment on a significant difference between the statistical R_t and the deterministic R_t from July 1 to the middle of December.**

   Response: Thank you so much for your observation. We made clarifications for this observation in Page 12, lines 405-414.

   Results and discussion (Page 12, lines 405-414)

   It is noteworthy however that at Q3, a difference between the R_t^d and R_t^s can be seen. As Q3 started, the initial conditions of the transmission dynamics were during a spike of cases yielding twice as much β value as Q2, hence as discussed in the results of elasticity indices, the spike in β also doubled the R_t^d. This implies that during the first data points in Q3, R_t^d captured the spike and the extreme values of R_t^s. Nevertheless, since R_t^s is data and time-dependent, its values decreased several days after day one of Q3 due to the perceived lag effect of the intervention implemented in that period. From here, it is conjectured that discrepancies between R_t^d and R_t^s in Q3 can be due to the assumed initial conditions (i.e., condition at day 1 of Q3) of the model. Nonetheless, the discrepancies between R_t^d and R_t^s are deemed tolerable.

7. **Figure 2 may be omitted. I think Figure 4 is enough.**

   Response: Thank you for this comment which helped avoid redundancy. We have omitted the said Figure 2 as suggested.

8. **I suggest using 10,000 samples in LHS-PRCC and 10,000 realizations in the bootstrapping.**

   Response: Thank you for this one. We have generated the new figures 2, 3, and 4 using 10,000 samples in LHS-PRCC and 10,000 realizations for the bootstrapping method. Please see the new descriptions of these figures as shown in the response for your third comment.

   Bootstrapping method (Page 7 Line 213-215):

   The parametric bootstrapping approach generated 10,000 bootstrap realizations assuming a Poisson error structure of the best fit from the LS fitting method.

Sensitivity analysis (Page 6 Line 181-183):
The method involved generating 10,000 samples via the LHS scheme to populate the LHS matrix. Each row on the LHS matrix was then passed to the SEI_HI_MR model to run 10,000 Monte Carlo simulations.

9. **Incorrect citation in line 186. There was no mention of LHS-PRCC in the works of Massey and Colleagues [42]. The authors may also add the link to the GitHub site.**

   Response: We are grateful for this observation. We admit a wrong citation for which we have now corrected to be Massey's github site herein cited at [43].

   References (Page 7, Reference 38)
   [38] Massey, S. C. Model Sensitivity Analysis. GitHub.com. 2019 Available from https://github.com/scmassey/model-sensitivity-analysis

10. **Figures 6 and 7 may be omitted. I think Figure 5 is enough.**

    Response: We appreciate this suggestion. We omitted these figures as suggested.

11. **Add a year on the xtick labels of Figure 8.**

    Response: Thank you for this suggestion. We have revised the x-axis tick labels of the former figure 8 to add a year. The new version is already presented as response to the third comment.

REFEREE 2

We thank you for your comments on the earlier version of this manuscript. In this version of the paper, we have done our best to address your suggestion. We appreciate your helpful suggestions and feel that it has vastly improved the manuscript.

Preliminary comments

Is the manuscript technically sound, and do the data support the conclusions?

The manuscript must describe a technically sound piece of scientific research with data that supports the conclusions. Experiments must have been conducted rigorously, with appropriate controls, replication, and sample sizes. The conclusions must be drawn appropriately based on the data presented.

- Partly. The methods employed are standard in disease modelling. The model formulation and parameter definitions are inconsistent in several instances, which could be important for the analysis and the interpretation of the analysis. The specifics are detailed in point number 1 in the below reviewer report.

  Response: We are very grateful for this remark. We have tried to address these referred inconsistencies in our revision of which the specific response is also detailed in point number 1.

- As the data has not been made available and is not publicly available, the study does not appear to be reproduceable.

  Response: Thank you so much for this comment. We now added a sample data similar to what we used in our run while not breaching the non-disclosure agreement we have signed along with our partner data provider. Please see the following edits.

  Data (Page 6, lines 138-155):
  Publicly available data on the daily COVID-19 new infected cases in the Philippines can be accessed through the government data drop [35]. However, the data on COVID-19 daily new infected cases in Davao City, Philippines, used in this study were obtained under a partnership and non-disclosure agreement from the Department of Health - Davao Center for Health Development (DOH - DCHD), Regional Epidemiology and Surveillance Unit (RESU). This partnership and data-sharing agreement were sought for better and more efficient data and result validation which would have been difficult if the data was acquired from the data drop. The epidemiological dataset used included dates of illness' onset, dates of surveillance report, and the health status upon admission of confirmed cases (e.g., asymptomatic, mild, moderate, severe, and critical) from March 8, 2020, to March 5, 2021. We categorized the epidemic data according to two groups: the infected individuals under monitoring in TTMFs (e.g., asymptomatic and mild cases), and the infected patients admitted to COVID-19 referral hospitals (e.g., moderate, severe, and critical cases). Even though the working model is a baseline model that does not distinguish vaccination as a disease control measure, the model could be used to assess pre-vaccination transmission dynamics. Hence, we only used data before the start of the vaccine rollout in Davao City, which was on March 5, 2021. A sample dataset which is in compliance with the non-disclosure agreement could be accessed through [36].

- The modelling results do support the conclusion; meanwhile, this linkage could be strengthened and the conclusions expanded. The conclusions do not explicitly reference the modelling analysis, could be an area of improvement. Point number 3 in the reviewer report below provides further details.

Response: Thank you so much for highlighting these points for the improvement of the paper. We have revised the results, discussions, and conclusions accordingly where we tried to explicitly cite all the necessary references, we've also made the reference as a hyperlink towards the section being referred to (see the revised Result and Discussion section and Conclusion section)

Results and Discussion: Qualitative analyses (Page 8 line 227-Page 9 line 302):
Some of the qualitative analyses performed in this study involved computing the basic (R_0) and time-varying deterministic reproduction numbers (R_t^d), and solving for the model's local stability, equilibrium points, and elasticity indices. The detailed steps to these analyses, proof and calculation can be found in the supplementary S1 File. The basic reproduction number (R_0) was the initial reproduction number of the pathogen at the start of the pandemic, whereas the deterministic time-varying reproduction number R_t^d was the reproduction number at any particular point in time t. The R_t^d described the average number of secondary infections caused by an infective individual in a susceptible population in the presence of control measures [38]. It provided a threshold for disease outbreak: if $R_t^d < 1$, the disease cannot persist; when $R_t^d \geq 1$, the virus can spread within the population where each infectious individual produced at least one new infection [46, 47]. The derived closed-form formulas of R_0 and R_t^d were as follows:

$$R_0 = \beta r \frac{\delta_M(1-q)}{AB} + \beta r \frac{\delta_H q B + \varphi_H m \delta_M (1-q)}{ABC} > 0,$$

$$R_t^d = \beta r \frac{\delta_M(1-q)S}{ABN} + \beta r S \frac{\delta_H q B + \varphi_H m \delta_M (1-q)}{ABCN},$$

$r, q \in [0,1]$ where $A = \psi(1-r) + \delta_H r q + \delta_M r(1-q) + \delta > 0$, $B = \varphi_H m + \gamma_M(1-m) + \delta > 0$, and $C = \mu_H + \delta + \gamma_H > 0$. Recall that A^{-1}, B^{-1}, and C^{-1} are just the mean residence time of compartments E, I_M, and I_H, respectively. This result meant that the average number of secondary infections caused by an infective individual is directly proportional to the transmission rate β and the positivity proportion r and is inversely proportional to A, and B.

To further determine how the parameters affect R_0, we solved for the elasticity index (normalized sensitivity index) [48]. The elasticity index measures the relative change of R_0 with respect to a parameter, for example, β, denoted by $\Upsilon_\beta^{R_0}$, such that

$$\Upsilon_\beta^{R_0} = \frac{\partial R_0}{\partial \beta} \times \frac{\beta}{R_0}.$$

The following are the proportionality and the elasticity indices for the relevant parameters:

The direct proportionality of the transmission rate β to R_0 is supported by its elasticity index of 1. This means that a unit increase of β results in a unit increase of R_0. Hence, any increase or decrease of the transmission of the pathogen from one person to another directly affects the size of the pandemic and its mitigation. Conversely, this result supports that the measures designed to hamper this transmission, such as wearing facemasks, hand washing, disinfection, observing physical distancing, and isolation, among others, [49, 50] can directly slow down the spread of the disease.

The direct proportionality of the positivity proportion r to R_0 is also quantified with an elasticity index of 1. This means that a unit increase in r also results in a unit increase of R_0. Thus, interventions designed to decrease r could directly lead to the mitigation of the spread of the disease. The interventions related to r are, but not limited to, contact tracing and testing. An increase in the effort in these interventions will directly increase the identified positive cases, thereby helps curb the pandemic for better management. That is, the more contact traced, quarantined, and tested individuals, the better, regardless if only a few among them become

a confirmed positive case. It is even more ideal to have a lower positivity rate among tested individuals.

The elasticity index of A on R_0 is -1. This backs the inverse proportionality of A to R_0, which means that a unit increase in A results to a unit decrease of R_0. Conversely, as A is just the inverse of the mean residence time in E (A^{-1}), a unit increase/decrease in A^{-1} would contribute to a unit increase/decrease in R_0, i.e., the faster the individual transitions out of the E compartment (smaller residence time), the more manageable the spread of the pathogen will be (with a reduced R_0). Since each term of A is an outflow rate identified with an intervention, this result lends support to more immediate COVID-19 test results combined with a reasonably shorter quarantine/isolation period (days) for reducing the risk of viral spread. However, due to the biological dynamics of COVID-19, there is a minimum required quarantine/isolation period, which imposes a positive lower bound for practical values of A when isolation is combined with limited testing capacity.

The inverse proportionality of B to R_0 is corroborated by its elasticity index of -0.99937. A unit increase of B would lead to a decrease in R_0 by 0.99937 units. Conversely, a unit increase in the mean residence time in the I_M compartment (B^{-1}) would lead to an increase of 0.99937 units to R_0. This signifies that the more number of asymptomatic and, or mildly symptomatic COVID-19 cases, the more the pandemic can be controlled. Hence, measures in ensuring that the vulnerable population gets due protection and medical attention produce positive results in preventing the further spread of the virus and disease-related deaths [51, 52].

The disease free, and endemic equilibrium points of the model were also solved and were verified by the proof of its local stability. This meant that two scenarios could be expected to come about in the COVID-19 pandemic dependent on certain conditions: (1) the disease would die out, or (2) becomes endemic [48]. These conditions were then verified to Davao City's circumstances, for which it was found that endemicity would, at best, be the more favorable scenario to be expected, as the disease free scenario is relatively too difficult to achieve, and at the point when this paper is written, is not yet realistic. Hence, policies toward the management of COVID-19 should consider an endemic scenario where the disease continues to exist but on a controlled manner. Recommendations relative to this are further expounded in the Conclusion part of this paper.

Epidemiological parameters (Page 11, lines 324-414):
From the gathered data, Fig 3 shows the COVID-19 daily and cumulative incidence in Davao City over the date of illness onset and the four-time periods that cover the community quarantine levels from March 8, 2020, to March 5, 2021. The complete list of system parameters as a result of the data fitting is presented in Table 2. Fig 3 showed the observed COVID-19 cases and the best model fit to the data and that the model was able to approximate the dynamics of the pathogen. Following the bootstrapping method, the resulting empirical distribution of parameters β and r, including R_t^d and the 95% confidence intervals of the estimated parameter values are presented in Fig 5. These show that the distribution is normal and that the model and the best fit values were able to capture the transmission dynamics of COVID-19 in Davao City, Philippines.

It was observed in the figures 3 5 that the estimated values of the transmission rate (β) were at its highest at the start of the pandemic and increased as the quarantine lockdowns were eased over time (compare Table 2). The values show that one infected individual transmits the disease every approximately $\dfrac{1}{\beta_{Q1}} = \dfrac{1}{0.2862} \approx 3.49$ days (83.86 hours) during the Q1 period, 7.79 days (186.92 hours) during Q2, 4.24 days (101.74 hours) during Q3, and 4.67 days (112.05 hours) during Q4. The effectiveness of the implemented CQ conform to a perceived lag effect [53], where the strictest measure implemented in ECQ (Q1) led to the least transmission rate at the next classification (Q2). However, since right after Q1 (ECQ; strictest), Q2 (GCQ; most permissive) was implemented, its

perceived lag effect brought about a higher transmission rate in Q3, which was almost twice as much as in Q2. The abrupt relaxation of quarantine classification from the strictest to the most relaxed one led to high transmissibility of the disease as more people were allowed mobility. Hence, it would have been best if a gradual relaxation of protocols were followed instead. Nevertheless, comparatively, these estimated values are similar and or within the range of published estimates throughout the world [23], for example, in India [51], and China [52]. Hence, the same as the case of China, with proper stringent preventive measures, the COVID-19 cases in Davao can still be brought down to manageable levels.

Table 2 show that the model estimates of the positivity proportion r were lowest during Q1 and highest during Q2. The results mean that 89.87% of the identified "close contacts" did not develop the disease during Q1, 82.42% during Q2, 82.98% during Q3, and 87.62% during Q4. As Davao City had strict mobility restrictions during Q1 may have led to lower contact among individuals and may have lessen the transmission of the pathogen, thus the tracing and testing capacities were more efficient hence the lowest r value. However, as lockdowns were eased in Q2 and Q3, we observed increases in r despite the tracing and testing efforts. This is due to the increase of the interacting population size while the tracing and testing capacity remained constant. Furthermore, the testing and tracing guidelines during Q2 and Q3 were a bit more relaxed causing a fewer identified "close contacts", hence the increase of r. The decrease of these values would have been a favorable outcome towards controlling the spread of the disease [28].

Different scenarios were considered concerning the different r values estimated as shown in Fig 4. It shows that if mass testing was done to at least halve the positivity rate, the daily new exposed population would have reduced from 73 to 21 individuals after 60 days of Q1 and from 34 to 14 individuals during Q2; the daily new I_M cases would have reduced from 24 to 5 individuals after 60 days of Q1 and from 30 to 8 individuals during Q2; and the daily new I_H cases would have become 0 from 2 individuals after 60 days of Q1 and 0 indv from 1 individuals during Q2. Moreover, if the efforts were a bit laxer to twice than what was the positivity rate, the daily new exposed population would have increased to 358 individuals after 60 days of Q1 and to 90 individuals during Q2, the daily new I_M cases would have increased to 175 individuals after 60 days of Q1 and to 122 individuals during Q2, while the daily new I_H cases would have become 13 individuals after 60 days of Q1 and 3 individuals during Q2. These results corroborate with the need for mass testing, contact tracing, and the need for faster release of test results towards mitigating the disease [54].

With respect to the severity proportion q as shown in Table 2, we found that 77.29% ($1-q = 0.7729$) of the confirmed cases are asymptomatic to mild, while 22.71% ($q = 0.2271$) were moderate to critical cases during Q1. Likewise, during Q2, 92.12% are asymptomatic to mild cases, and 7.88% are moderate to critical cases. The moderate to critical cases during Q3 slightly increased with 8.42% of the confirmed cases and 7.42% moderate to critical cases during Q4. The majority of the confirmed cases in Davao City consisted of asymptomatic to mild cases. However, even though the ratio of moderate to critical cases in Davao to asymptomatic to mild cases is relatively low, its total number of cases still amounts to more than Davao City's total COVID-19 bed capacity. Hence, Davao COVID-19 referral hospitals are usually maxed out of their capacities [55]. Policies and budgetary support towards the increase of Davao City's health system and increasing hospital capacities should be prioritized.

Table 2 further show that in Davao City, a COVID-19-induced death (represented by μ_H) occurs every approximately $\dfrac{1}{\mu_H} = 8.17$ days in Q1, 6.33 days in Q2, 3.42 days in Q3, and 3.33 days in Q4. Meanwhile, based on the data, the hospitalized cases are discharged at the rate (γ_H) of 21.5 days in Q1, 20.5 days in Q2, 17 days in Q3, and 20 days in Q4. The infectious period of moderate-critical

cases shortens during Q3 as the number of hospitalized individuals eventually reached hospital capacity, which, in turn led to a faster COVID-19-induced death rate [55].

As seen in Fig 6, the overall trend of the deterministic reproduction number R_t^d follows that of the statistical reproduction number R_t^s. Furthermore, there is a decrease in the time-varying reproduction number during the Q2 period as compared to Q1. This is during the early stages of the pandemic and the community quarantine intervention has shown to have a positive effect in mitigating the spread of the disease. However, when the government attempted to ease the economic burden by implementing the MGCQ in Q3, the R_t^d increased indicating a faster disease spread in the city. The reimplementation of GCQ in Q4 has reduced the R_t^d to a value of slightly less than one.

Conclusion (Page 13, lines 427-469):
The result on the reproduction numbers (R_0 and R_t^d) and elasticity indices as discussed in the Qualitative analysis subsection implied that the number of secondary infections was driven by the number of infectious populations and how often they interact with the susceptible population. The transmission rate in Davao City was estimated to be highest during ECQ, i.e., at the start of the pandemic (Table 2). This indicated that Davao City may had limited and insufficient resources against the emergence of a pandemic. Hence, there is a need to better equip the healthcare system and the local government units (LGU) for better disaster management and prevention of future pandemics. A multi-sectoral framework, policies, and protocols of how the different agencies could collaboratively work should already be put in place even before the occurrence of any health emergencies. The National, Regional, Provincial, City, and Municipal Disaster Risk Reduction and Management Offices, the Department of Health (DOH), the Department of Interior and Local Government, the LGU, Provincial, City, Municipal, and Barangay Health Offices are some of the core agencies necessary for effective pandemic management. Guides, manuals, and protocols should be collaboratively drafted by these agencies and periodic capacity training for its personnel should be incorporated into its mandate for better preparedness and mitigation. Based on the model results, policies toward the reduction of the transmission rate such as the implementation of containment measures, availability and wearing of protective equipment, and sanitation are some of the key factors to consider.

On the other hand, as discussed in the above subsection on Epidemiological parameters and corroborated by Table 2 concerning the transmission rates, an abrupt change from a strict containment measure to a lax one could negatively affect the control measures implemented especially because of a perceived lag effect. A protocol for a gradual easement of containment measures should be implemented instead. Moreover, since we know that the results with respect to the positivity proportion r corroborated the need to put forth better monitoring and identification protocols which include, but are not limited to, testing and contact tracing capabilities, and travel and mobility surveillance. Furthermore, the results for the severity proportion q suggested the need to allocate funding and to study the appropriate ratio of the hospital carrying capacity to the city population because despite the small number of severely symptomatic COVID-19 patients, the hospitals in Davao City still were overwhelmed. More than allocating funds for equipment and buildings, the production of and adequate remuneration for an appropriate number of nursing and healthcare professionals should be a necessity [56]. The need for this action was further supported by the results with respect to the disease-induced death rate μ_H as it only critically increased in Q4 when many of the hospitals reached its maximum capacity, and many of the healthcare workers were overwhelmed by the number of hospital admissions. Despite having available hospital beds, the number of attending nurses and doctors was inadequate, and the available ones are already highly exhausted. Government interventions such as the provisions of scholarships, opening of job positions, and offering of competitive remuneration among others should have more investments and be made more accessible to all aspiring healthcare workers.

Has the statistical analysis been performed appropriately and rigorously?

- 3 Yes, the statistical analysis performed in the manuscript (1. LHS/PRCC and 2. Uncertainty quantification via assuming Poisson error structure in the best fit incidence and refitting to each realization for the downstream analysis) are performed appropriately and rigorously.

  Response: We are very grateful for this remark. Thank you so much! In this revision, we have reperformed these analyses and expanded the sample size from 2,000 to 10,000 while generating a more appropriate figure.

Have the authors made all data underlying the findings in their manuscript fully available?

The PLOS Data policy requires authors to make all data underlying the findings described in their manuscript fully available without restriction, with rare exception (please refer to the Data Availability Statement in the manuscript PDF file). The data should be provided as part of the manuscript or its supporting information, or deposited to a public repository. For example, in addition to summary statistics, the data points behind means, medians and variance measures should be available. If there are restrictions on publicly sharing data—e.g. participant privacy or use of data from a third party—those must be specified.

- No, information about the data acquisition appears in the manuscript on L149 – 152. In my experience, it is standard for such linelisted epidemiological data (i.e., individual confirmed case data) to be private and not able to share publicly. While the linelist may be private, it might be possible to share the time series extracted from the linelist.

  Response: We appreciate your observation in this regard. However, we regret to say that the extracted time series are still covered in the nondisclosure agreement between the research team and the data provider. Nevertheless, a dataset for public use is also available. So, we are providing this as the sample in the repository as stated in the response to the general comment.

  Materials and methods: Data (Pages 6 Line 136-155):
  Publicly available data on the daily COVID-19 new infected cases in the Philippines can be accessed through the government data drop [35]. However, the data on COVID-19 daily new infected cases in Davao City, Philippines, used in this study were obtained under a partnership and non-disclosure agreement from the Department of Health - Davao Center for Health Development (DOH - DCHD), Regional Epidemiology and Surveillance Unit (RESU). This partnership and data-sharing agreement were sought for better and more efficient data and result validation which would have been difficult if the data was acquired from the data drop. The epidemiological dataset used included dates of illness' onset, dates of surveillance report, and the health status upon admission of confirmed cases (e.g., asymptomatic, mild, moderate, severe, and critical) from March 8, 2020, to March 5, 2021. We categorized the epidemic data according to two groups: the infected individuals under monitoring in TTMFs (e.g., asymptomatic and mild cases), and the infected patients admitted to COVID-19 referral hospitals (e.g., moderate, severe, and critical cases). Even though the working model is a baseline model that does not distinguish vaccination as a disease control measure, the model could be used to assess pre-vaccination transmission dynamics. Hence, we only used data before the start of the vaccine rollout in Davao City, which was on March 5, 2021. A sample dataset which is in compliance with the non-disclosure agreement could be accessed through [36].

Is the manuscript presented in an intelligible fashion and written in standard English?

PLOS ONE does not copyedit accepted manuscripts, so the language in submitted articles must be clear, correct, and unambiguous. Any typographical or grammatical errors should be corrected at revision, so please note any specific errors here.

- The manuscript is overall presented in an intelligible fashion. The authors might consider streamlining the main text and placing non-critical material in an appendix. The English writing and scientific writing could be improved (point number 4 in the below reviewer report). Given the number of typos and grammatical errors, the authors might consider thorough proofreading and editing for the journal readership. Additionally point number 7 in the below reviewer report notes several instances of potential consideration.

  Response: We gratefully welcome your suggestions. Some analyses are now moved to the appendix to hopefully streamline the manuscript. We have also subjected the revised paper under grammarly check for both plagiarism and writing issues. The person acknowledged in the manuscript also proofread the paper. All notes in point number 7 have been appropriately reflected in this version. Thank you so much!

- The manuscript proposes a modified SEIR-type disease transmission model, formulated as a deterministic system of ordinary differential equations, which accounts for several public health measures aimed at mitigating the transmission of SARS-CoV-2. The model was fitted to four phases of interventions in Davao City, Philippines (so called community quarantines), prior to the rollout of vaccination to the population. The model fitting yielded parameter estimates according to each phase of public health interventions. A sensitivity analysis using the Latin hypercube sampling / partial rank correlation coefficient (LHS/PRCC) methodology was performed. Elements of retrospective assessment of the model fit according to different phases of public health measures in Davao City were included. Additionally, several recommendations in terms of public health policy to mitigate transmission of SARS-CoV-2 were provided based on the analysis. The manuscript confronts a relevant scientific problem of the (retrospective) assessment or evaluation of different public health interventions aimed at mitigating transmission of SARS-CoV-2. The topic of intervention assessment in the context of SARS-CoV-2 and other respiratory pathogens is of importance, given the burden of disease and the general need for quantitative evidence to inform policy- and decision- making. I have concerns regarding the model formulation and its novelty. Likewise, the manuscript would benefit from thorough editing for scientific writing and English. Lastly, the linkage between the modelling analysis conducted and public health policy recommendation could be strengthened and, ideally, expanded. Overall, the manuscript would benefit from a revision to address and clarify these points.

  Response: We greatly appreciate this thorough and profound assessment. We have tried to address the concerns on the model formulation and explicitly indicated the limitations of the current study. We have also expounded on the linkage between the analyses and the policy recommendations (which were consulted to and verified with the co-authors from the Philippines' Department of Health Davao Region). This version was also subjected under grammarly services and was proofread for better readability. Thank you so much for the suggestions! We are hoping that the edits are satisfactory.

Major comments

Regarding the model (1) formulation and its novelty:

1. The model formulation:

   (a) Several parameter definitions are inconsistent with the model formulation. This may be important for both the analysis and the interpretation of the analysis.

   - The mean residence time in the E compartment, determined by all relevant outflows, is $\psi(1-r)+\delta_H rq+\delta_M r(1-q)+\delta)^{-1}$ days. Meanwhile, each removal process has an associated (and different) length of stay in E listed in the description – in contradiction with

the model specification. As an example, the quarantine period is written and interpreted in the manuscript as ψ^{-1} days, the latency periods as δ_M^{-1} and δ_H^{-1} days. However, the mean residence time in E is determined as $\psi(1-r)+\delta_H rq+\delta_M r(1-q)+\delta)^{-1}$ and will be the same for all individuals entering E, regardless of the compartment to which an individual is transferred to. The same issue is present in the I_H and I_M compartments, which may also be important, as the dwell time distributions in those compartments determine the distribution of the infectious period. The mean residence time in I_M (mean infectious period) is $(\varphi_H m+\gamma_M(1-m)+\delta)^{-1}$ days. The mean residence time in I_H (mean infectious period) is $(\mu_H+\delta+\gamma_H)^{-1}$ days. It is possible to resolve this issue where the residence times are determined as intended; however, it appears to require either the model structure to be revisited or the model parameterized differently.

Response: Thank you so much for this spot-on comment. We have revised some of the parameter descriptions for consistency and included the description and calculation of the mean residence times for E, I_M, and I_H (i.e A, B, and C, respectively). We have also linked these to some relative analyses and discussions. Thank you for this suggestion!

Model formulation (Page 4, lines 119-125):

The schematic diagram in Fig 1 depicted the disease transmission dynamics, with the corresponding parameter descriptions, range of values, and sources, were detailed in Table **??**. Herein, the mean residence time in E, in I_M, and in I_H are respectively given as
$A^{-1}=(\psi(1-r)+\delta_H rq+\delta_M r(1-q)+\delta)^{-1}$,
$B^{-1}=(\varphi_H m+\gamma_M(1-m)+\delta)^{-1}$, and
$C^{-1}=(\mu_H+\delta+\gamma_H)^{-1}$.

Table 3: **Parameter description, values, and sources for Davao City, Philippines.**

Parameter	Description	Value/Range	Unit	Source
N	Total Population	1,816,987	indv	[33]
β	Transmission rate	$[0, 10]$	per day	Estimated
r	Proportion of Exposed that becomes infectious	$[0, 1]$	% per day	Estimated
δ_H	$E \to I_H$: Reciprocal of the period from onset of illness to Hospital Admission	$[0, 1]$	per day	*
δ_M	$E \to I_M$: Reciprocal of the period from onset of illness to TTMF Admission	$\frac{1}{3}$,$[0, 1]$	per day	*
γ_H	$I_H \to R$: Recovery rate of I_H individuals	$[0, 1]$	per day	*
γ_M	$I_M \to R$: Recovery rate of I_M individuals	$[0, 1]$	per day	*
μ_H	$I_H \to D$: COVID-19 related death rate	$[0, 1]$	per day	*
q	Proportion of Reported cases that are Moderate-Critical	$[0, 1]$	% per day	*
m	Proportion of I_M cases that become Critical/Severe	$[0, 1]$	% per day	*
ψ	Discharging rate of Exposed	$\frac{1}{14}$	per day	[14]
φ_H	$I_M \to I_H$: Hospitalization rate of I_M	$\frac{1}{5}$	per day	*
δ	Natural Death Rate	$\frac{1}{26017.2}$	per day	[34]
α	Recruitment Rate	69.8379**	per day	[34]
A^{-1}	Mean residence time in E	$[0, 20]$	days	Calculated
B^{-1}	Mean residence time in I_M	$[0, 20]$	days	Calculated
C^{-1}	Mean residence time in I_H	$[0, 20]$	days	Calculated

* estimated from the data, **$\alpha \approx N\delta$

Qualitative analyses (Page 8, lines 239-291): The derived closed-form formulas of R_0 and R_t^d were as follows:

$$R_0 = \beta r \frac{\delta_M(1-q)}{AB} + \beta r \frac{\delta_H q B + \varphi_H m \delta_M (1-q)}{ABC} > 0,$$

$$R_t^d = \beta r \frac{\delta_M(1-q)S}{ABN} + \beta r S \frac{\delta_H q B + \varphi_H m \delta_M (1-q)}{ABCN},$$

$r, q \in [0, 1]$ where $A = \psi(1-r) + \delta_H r q + \delta_M r(1-q) + \delta > 0$, $B = \varphi_H m + \gamma_M(1-m) + \delta > 0$, and $C = \mu_H + \delta + \gamma_H > 0$. Recall that A^{-1}, B^{-1}, and C^{-1} are just the mean residence time of compartments E, I_M, and I_H, respectively. This result meant that the average number of secondary infections caused by an infective individual is directly proportional to the transmission rate β and the positivity proportion r and is inversely proportional to A, and B.

To further determine how the parameters affect R_0, we solved for the elasticity index (normalized sensitivity index) [48]. The elasticity index measures the relative change of R_0 with respect to a parameter, for example, β, denoted by $\Upsilon_\beta^{R_0}$, such that

$$\Upsilon_\beta^{R_0} = \frac{\partial R_0}{\partial \beta} \times \frac{\beta}{R_0}.$$

The following are the proportionality and the elasticity indices for the relevant parameters:

i. The direct proportionality of β to R_0 is supported by its elasticity index of 1. This means that a unit increase of β results in a unit increase of R_0. Hence, any increase or decrease of the transmission of the pathogen from one person to another directly affects the size of the pandemic and its mitigation. Conversely, this result supports that the measures designed to hamper this transmission, such as wearing facemasks, hand washing, disinfection, observing physical distancing, and isolation, among others, [49, 50] can directly slow down the spread of the disease.

ii. The direct proportionality of r to R_0 is also quantified with an elasticity index of 1. This means that a unit increase in r also results in a unit increase of R_0. Thus, interventions designed to decrease r could directly lead to the mitigation of the spread of the disease. The interventions related to r are, but not limited to, contact tracing and testing. An increase in the effort in these interventions will directly increase the identified positive cases, thereby helps curb the pandemic for better management. That is, the more contact traced, quarantined, and tested individuals, the better, regardless if only a few among them become a confirmed positive case. It is even more ideal to have a lower positivity rate among tested individuals.

iii. The elasticity index of A on R_0 is -1. This backs the inverse proportionality of A to R_0, which means that a unit increase in A results to a unit decrease of R_0. Conversely, as A is just the inverse of the mean residence time in E (A^{-1}), a unit increase/decrease in A^{-1} would contribute to a unit increase/decrease in R_0, i.e., the faster the individual transitions out of the E compartment (smaller residence time), the more manageable the spread of the pathogen will be (with a reduced R_0). Since each term of A is an outflow rate identified with an intervention, this result lends support to more immediate COVID-19 test results combined with a reasonably shorter quarantine/isolation period (days) for reducing the risk of viral spread. However, due to the biological dynamics of COVID-19, there is a minimum required quarantine/isolation period, which imposes a positive lower bound for practical values of A when isolation is combine with limited testing capacity.

iv. The inverse proportionality of B to R_0 is corroborated by its elasticity index of -0.99937. A unit increase of B would lead to a decrease in R_0 by 0.99937 units. Conversely, a unit increase in the mean residence time in the I_M compartment (B^{-1}) would lead to an increase of 0.99937 units to R_0. This signifies that the more number of asymptomatic and, or mildly symptomatic COVID-19 cases, the more the pandemic can be controlled. Hence, measures in ensuring that the vulnerable population gets due protection and medical attention produce positive results in preventing the further spread of the virus and disease-related deaths [51, 52].

(b) The stratification of the infectious population and the assumptions about the infectivity of individuals of different statuses would be good to justify, discuss as limitations, and/or revisit. Specifically,

- In the current model, undetected cases (whether presymptomatic, symptomatic or asymptomatic) do not contribute to the force of infection (FOI) whereas those PCR positive cases (I_M and I_H) do contribute to the FOI. Given IPC measures in the hospital setting, one might expect to contribute relatively less to the force of infection, compared to undetected infections in the community. Likewise, those mild or asymptomatic cases which have been diagnosed (PCR positive) – individuals in the compartment – one would expect are ordered to isolate following their diagnosis and therefore contribute little to the FOI compared to undetected cases. Is it true that individuals who have tested positive are required to isolate in the jurisdiction considered (Davao city)? Overall, the authors might consider adding a compartment for infectious and undetected cases (not PCR positive).

Response: We gratefully acknowledge the relevance of this comment. Thank you! We have specified the limitations of the current study in Page 3 lines 55-78 justifying some of the assumptions relative to Davao City's circumstances. The points raised have really been considered but are being studied in another on-going research (extension of this baseline paper) accounting for the underreporting, differing contributions to the transmission, and optimal control. However, these matters are beyond the scope of the current study. Hence, we opted to add these to limitations and recommendations.

Introduction (Page 3 lines 55-78):

This paper modified the classic Susceptible-Exposed-Infected-Recovered ($SEIR$) deterministic compartmental model [1] into a Susceptible-Exposed-Infectious Hospitalized-Infectious Monitored-Recovered (SEI_HI_MR) model to describe the dynamics of COVID-19 in Davao City incorporating the Philippines' Department of Health's quarantine and isolation protocols, namely quarantining exposed individuals and isolating confirmed positive cases according to the severity of symptoms or presence of comorbidity [14]. This model incorporated an outflow from the E compartment back to S similar to [32]— a dynamic that has not been widely applied to models in a Philippine regional setting. This link signified that, instead of the standard classification of the E compartment [1], not all exposed individuals in this model are on a latent stage of the disease, do not become infectious, and hence become susceptible again after they were released from quarantine or isolation. Moreover, the model also incorporates the progression or worsening of symptoms of some previously asymptomatic positive cases (*i.e.,* presymptomatic). Disease-related deaths also in this model are assumed to only occur among severely symptomatic patients. Furthermore, in Davao City's context, isolation and, or quarantining of the infectious individuals do not guarantee that the patient has stopped contributing to the pathogen's transmission; viral spread may occur before an intervention due to delays in test result. Moreover, health and emergency workers can still get infection from quarantined or isolated individuals. Hence in this model, we assumed that the compartments I_H and I_M equally drive the transmission regardless of isolation or quarantine. Nevertheless, the contact tracing method in Davao City during the period covered in this paper is assumed to be efficient whereby minimizing the possibility of an untraced infectious individual. Thus, the model does not incorporate a compartment for undetected cases to make analysis tractable.

Conclusions (Page 14, Lines 476-486):

Moreover, researchers and practitioners must be aware of the limitations of compartmental-based modeling (SIR, SEIR, etc.). This approach generally assumes that the community under infection is homogeneous: each human host infects and undergoes infection in the same manner. A comparison of our analysis with agent-based models [57] can reveal additional factors that can refine epidemic dynamics and projections. Since our model assumed data prior to the vaccine rollout in Davao City, a suitable refinement of our model should incorporate community-wide inoculation dynamics. Other possible extensions account for risk groups [58], particularly age groups [59], and untraced and undetected infectious individuals in the context of testing and contact tracing [60, 54].

(c) Is any aspect of under-reporting or under-ascertainment being considered? In the context of SARS-CoV-2, a substantial proportion of infected individuals typically remain undetected by the surveillance system; however, the model nor the data fitting process does not appear to account for this. Is this not the case in Davao City? This may be particularly relevant for the mild or asymptomatic cases.

Response: Thank you very much for this insight. To keep our model tractable to analysis, we did not account for under-reported or under-ascertained cases, assuming an effective contact

tracing method. We clarify this in lines 121-125 in our revision .
Introduction (Page 3 Lines 75-78):

Nevertheless, the contact tracing method in Davao City during the period covered in this paper is assumed to be efficient whereby minimizing the possibility of an untraced infectious individual. Thus, the model does not incorporate a compartment for undetected cases to make analysis tractable.

2. It is claimed in the manuscript that the model is unique in the context of existing publications in that there is a linkage between E back to S, modelling the release from quarantine among individuals who were not infected. The quarantine and contact tracing process has been modelled in a number of published SARS-CoV-2 transmission dynamics models (e.g., reference [1], see Section 2.2 and Figure 2).

   1 Tang B, Wang X, Li Q, Bragazzi NL, Tang S, Xiao Y, Wu J. Estimation of the transmission risk of the 2019-nCoV and its implication for public health interventions. Journal of Clinical Medicine. 2020 Feb 7;9(2):462.

   Response: We are grateful for this comment. We have revised the manuscript accordingly and added this publication among the cited references.
   Introduction (Page 3, Lines 61-66):

   This model incorporated an outflow from the E compartment back to S similar to [32]— a dynamic that has not been widely applied to models in a Philippine regional setting. This link signified that, instead of the standard classification of the E compartment [1], not all exposed individuals in this model are on a latent stage of the disease, do not become infectious, and hence become susceptible again after they were released from quarantine or isolation.

3. Regarding the relevance of the analysis for public health policy decision-making:

   - The linkage between the utility of the modelling analysis to support relevant policy- and decision- making and the claims in introduction could benefit from strengthening. Specifically, clear and actionable control guidance based on the analysis conducted, with a reference to the relevant elements of analysis (Figures, Tables, etc.) would be ideal. Control guidance has been provided to some extent in L395 – 420; but, should reference the modelling analysis and ideally be expanded. A demonstration or illustration as to how the modelling results can be utilized to inform PH policy may also allow for the adoption and application of the framework by external users.

     Response: We appreciate your comments in this regard on relevance of analysis for public health policy decision-making. We elaborated our model-driven recommendations in the Conclusion Section as displayed as response to other comments.

4. The manuscript could be clearer and more concise. The manuscript would benefit from thorough editing for scientific writing and English writing.
   Response: We acknowledge that there still are room for the improvement of the contents written in the current revision. We have subjected the revised paper under grammarly check for both plagiarism and writing issues. The person acknowledged in the manuscript also proofread the paper.

5. Limitations of the analysis do not appear to be present in the manuscript. Limitations of the analysis would be good for the authors to include in the manuscript (typically included in Discussion).
Response: We greatly appreciate this comment and have improved the last paragraph of the discussion to highlight some of the limitations of the analysis. In lines 476-486 of the revised manuscript, we identified a common limitation of SEIR-based modeling, namely a homogeneous host community, recommending an agent-based approach as a possible future direction. We also identified the following extensions to our model: vaccination dynamics, risk groups, testing and contact tracing.
Conclusion (Page 12, Lines 476-486):
Moreover, researchers and practitioners must be aware of the limitations of compartmental-based modeling (SIR, SEIR, etc.). This approach generally assumes that the community under infection is homogeneous: each human host infects and undergoes infection in the same manner. A comparison of our analysis with agent-based models [57] can reveal additional factors that can refine epidemic dynamics and projections. Since our model assumed data prior to the vaccine rollout in Davao City, a suitable refinement of our model should incorporate community-wide inoculation dynamics. Other possible extensions account for risk groups [58], particularly age groups [59], and untraced and undetected infectious individuals in the context of testing and contact tracing [60, 54].

6. L327 – 328 and Table 1: Were these parameter values reported in Table 1 estimated from fitting to or to (or both)? It would be good to specify the objective function and explain adequately the process. If applicable, the authors might consider also including parameter tables for both fitting results, allowing for comparison of estimates using different data streams
Response: We have included the following statements in the manuscript. Please review Page 7, Lines 196-203
Model parameterization (Page 7, Lines 196-203):
To fit the asymptomatic-or-mild daily incidence data to the model, we defined the newly infected people under monitoring as $M(t) = \delta_M r(1-q)E$. The moderate-to-critical daily incidence data were fitted to the model using the new hospitalized infected people defined as $H(t) = \delta_H rqE$. The data-fitting problem was then solved using the least squares (LS) technique given by the objective function $f\hat{(t_i)} = argmin \Sigma_{i=1}^{n} (f(t_i) - y(t_i))^2$, $y(t_i)$ was the observed daily new cases, and $f(t_i)$ was the corresponding model simulation. In particular, $f(t_i) = \big(M(t_i), H(t_i)\big)$ and $y(t_i)$'s are the daily reported monitored and hospitalized cases.

7. Several instances of suspected typos and phrasing/terminology that the authors may consider revising in the manuscript are included below. A few suggestions the authors may consider are included:

   (a) L6: The word "practice" may be replaced with "implementation".
   Response: Thank you for this suggestion. We have replaced it as recommended.

   (b) L6 and throughout the text: non-pharmaceutical interventions are generally abbreviated as 'NPIs'.
   Response: Thank you for noticing! We have revised all instances as suggested.

   (c) L19 – 38: The description of the four phases of control measures could be clearer. The authors may consider defining the phases in the order in which they were implemented chronologically (for example) or include a table with their definitions.
   Response: We appreciate this recommendation. We have added table 1 with a few of the apparent differences in implementation across quarantine classifications.

   Introduction Table 1):

Table 4: **Quarantine classification, sample restrictions, Davao City, Philippines [15].**

	**Quarantine Classification**			
	**ECQ**	**MECQ**	**GCQ**	**MGCQ**
Population	100% stay at home	100% stay at home	Elderly & youth	Permissive socio-economic activities with minimal public health standards
Gathering	Not Allowed	Restricted to 5 max	Restricted to 10 max	
Travel	No public transport; no domestic flights; limited international flights	No public transport; limited international flights; controlled in-bound travels	Public transport with strict distancing	
Government	Skeletal onsite; Others work from home	Skeletal onsite; Others work from home	Alternative work arrangement; 4-day workweek	

(d) L81 and after: traditionally Materials and methods, Results, Discussion and Conclusion are written in past tense.
Response: We are grateful for this comment. We have changed the tenses in these sections as recommended. Thank you.

(e) L96: Suspected typo. "homogenous" may read "homogeneous".
Response: Thank you for noticing. We have now corrected this typological error. Thank you!

(f) L129 – 134: The discussion about the uniqueness of the model would generally not be an element of Methods, but of Introduction. Methods typically includes a concise and streamlined description of the methodology employed.
Response: Thanks you for pointing this out. We have moved the uniqueness and the structure of the model in the introduction section Lines 55-78.

(g) L134 – 135: The cases mentioned could be considered as presymptomatic, i.e., those individuals who have not yet developed symptoms but eventually will develop symptoms.
Response: Thank you for the reminder! We now added this in the description. Please see line 68
Introduction (Page 3, Lines 66-68):
Moreover, it also incorporates the progression or worsening of symptoms of some previously asymptomatic positive cases (*i.e.*, presymptomatic).

(h) L177: Suspected typo. "as priori" may read "a priori".
Response: Thank you for this! We have now corrected this part of the manuscript. Please see line 171
Sensitivity analysis (Page 6, Lines 171):
Sensitivity analysis was performed *a priori* to parameter estimation.

(i) L210 – 211: The phrasing statistically solved seems nonstandard. The parameter estimation process from data is generally referred to as estimation. The alternative "Other parameters were either estimated from the data [37] or taken from existing published literature" might be clearer.
Response: We are grateful for this point. This has been duly implemented. Tables 2 and 3 have also been revised/provided for better clarity.

(j) L271: The authors may consider revising this line. From context, it appears the authors may instead be referring to a unit increase in the parameter which is related to contact tracing.
Response: We warmly welcome this suggestion. Page 9 Lines 270-282 have been revised accordingly.
Qualitative analyses (Page 9, Lines 270-282):
The direct proportionality of the positivity proportion r to R_0 is also quantified with an elasticity index of 1. This means that a unit increase in r also results in a unit increase of R_0. Thus, interventions designed to decrease r could directly lead to the mitigation of the spread of the disease. The interventions related to r are, but not limited to, contact tracing and testing. An increase in the effort in these interventions will directly increase the identified positive cases, thereby helps curb the pandemic for better management. That is, the more contact traced, quarantined, and tested individuals, the better, regardless if only a few among them become a confirmed positive case. It is even more ideal to have a lower positivity rate among tested individuals.

(k) L395-396: The transmission rate cannot be observed directly – only inferred or estimated. This may more appropriately read: '. . . the transmission rate in Davao City was estimated to be highest during MGCQ. . . ', or equivalent.
Response: Thank you for the comment. We have revised all instances accordingly.

(l) L403: Suspected typo. "closed contacts" → "close contacts".
Response: Thank you for noticing! We have accordingly corrected this line.

(m) L395 – 420: When referring to the analysis within, reference the appropriate Table or Figure, so as to support control guidance with the modelling analysis.
Response: Thank you for pointing this out. To guide the readers in the analysis part, we added the appropriate referencing for the lines mentioned above in our revised paper.

(n) L395-396: this can be stated more concisely by referencing the expression for the reproduction number.
Response: Thank you for this. We have incorporated this suggestion in the revised paper.

(o) The authors might consider to place non-critical elements of the main text and figures in an appendix so as to streamline the main text (e.g., the standard mathematical analysis beginning on L329 could be appendix material).
Response: We warmly appreciate this recommendation. We have moved the referenced lines from the original text to the appendix material. We have accordingly revised Page 8 lines 228-302 to streamline the contents of the manuscript as suggested.

8. The quality of Figures 2, 4, 5, 6, 7 and 8 should be increased to publication quality.
Response: Thanks a lot for this comment. We reran all analyses for the current revision (more samples, etc) and generated some revised figures. We have also reviewed and tried to conform to the journal's guidelines for figures. Some of the redundant figures were also omitted as recommended by the other reviewer. Please see the new figures.

9. Figure 3. The time-dependent sensitivity analysis may be interpreted in terms of retrospective intervention assessment and public health policy. This may be an element the authors could potentially

elaborate upon.

Response: Interpretation of sensitivity analysis with regards to policy recommendations have been included as follows:

Sensitivity analyses (Page 10, Lines 308-3222):

In addition to β and r parameters, δ_M was also strongly positively correlated to I_M cases. These indicated a strong association between high values of the β, r, δ_M parameters to the number of COVID-19 cases, specifically during the early stages of the pandemic. The correlation of these parameters to the response functions decreased over time during the course of the pandemic. This result supported the need to distinguish these three (3) parameters as essential factors in the transmission dynamics of the virus. However, as δ_M is dependent on the biology of SARS-CoV-2, intervention recommendations focused mainly on policy-preventable parameters β and r. Moreover, as the severity proportion q and disease-induced death rate μ_H directly equates to COVID-19 morbidities that we also put attention to these parameters. The implementation of control interventions were essential for the reduction of the values of these parameters (β, r, q, and μ_H) to mitigate the spread and lessen the hospitalizations due to COVID-19. Logrosa and colleagues corroborate that if the local government prioritized reducing COVID-19 fatality, the COVID-19 pandemic will be manageable, at least in the context of Davao City, Philippines [28].

10. The parameter can be interpreted as the transmission probability per contact multiplied by the daily per capita contact rate. This interpretation of the estimated transmission coefficients could enter the modelling analysis and subsequent public health policy recommendation (e.g., entering the Discussion in terms of policies based on the corresponding estimates of in each phase). This could add another dimension to the analysis, given that behavior and thus contact rates change temporally as well as the probability of transmission upon contact.

    Response: Thank you for this comment. The response in third preliminary comment encompasses our action taken relative to this suggestion.

11. L251 – 252, regarding point number 1 concerning the residence times specified in the model: The inverse of the mean residence times in the compartments can be observed in the calculation of the reproduction number (the parameters defined as A, B and C).

    Response: Thank you so much for this suggestion. We have included this comment in consideration as we acted on point number 1. Relative edits have already been reflected in the revised version.

References

[1] Anderson RM, May RM. Infection diseases of humans. Dynamics and control. Oxford and New York: Oxford University Press. 1991.

[2] Lu H, Stratton CW, Tang YW. Outbreak of pneumonia of unknown etiology in Wuhan, China: The mystery and the miracle. Journal of medical virology. 2020 Apr;92(4):401.

[3] Riou J, Althaus CL. Pattern of early human-to-human transmission of Wuhan 2019 novel coronavirus (2019-nCoV), December 2019 to January 2020. Eurosurveillance. 2020 Jan 30;25(4):2000058.

[4] Ding Q, Lu P, Fan Y, Xia Y, Liu M. The clinical characteristics of pneumonia patients coinfected with 2019 novel coronavirus and influenza virus in Wuhan, China. Journal of medical virology. 2020 Sep;92(9):1549-55.

[5] Huang C, Wang Y, Li X, Ren L, Zhao J, Hu Y, Zhang L, Fan G, Xu J, Gu X, Cheng Z. Clinical features of patients infected with 2019 novel coronavirus in Wuhan, China The lancet. 2020 Feb 15;395(10223):497-506.

[6] Pei S, Kandula S, Shaman J. Differential effects of intervention timing on COVID-19 spread in the United States. Science advances. 2020 Dec 1;6(49):eabd6370.

[7] Inchausti F, MacBeth A, Hasson-Ohayon I, Dimaggio G. Psychological intervention and COVID-19: what we know so far and what we can do. Journal of contemporary psychotherapy. 2020 Dec;50:243-50.

[8] Sun Q, Qiu H, Huang M, Yang Y. Lower mortality of COVID-19 by early recognition and intervention: experience from Jiangsu Province. Annals of intensive care. 2020Dec;10(1):1-4.

[9] Tay MZ, Poh CM, Rénia L, MacAry PA, Ng LF. The trinity of COVID-19: immunity, inflammation and intervention. Nature Reviews Immunology. 2020 Jun;20(6):363-74.

[10] Rastogi S, Pandey DN, Singh RH. COVID-19 pandemic: A pragmatic plan for ayurveda intervention. Journal of Ayurveda and Integrative medicine. 2020 Apr 23.

[11] Li Y, Zhang R, Zhao J, Molina MJ. Understanding transmission and intervention for the COVID-19 pandemic in the United States. Science of the Total Environment. 2020 Dec 15;748:141560.

[12] Inter-Agency Task Force for the Management of Emerging Infectious Diseases [IATF]. Omnibus guidelines on the implementation of community quarantine in the Philippines, Resoloution No 38. Amendments May 22, 2020. 2020. Available from: https://doh.gov.ph/sites/default/files/health-update/IATF-Resolution-No.-38.pdf

[13] Shim E, Tariq A, Choi W, Lee Y, Chowell G. Transmission potential and severity of COVID-19 in South Korea. International Journal of Infectious Diseases. 2020 Apr 1;93:339-44.

[14] Department of Health [DOH]. Department Memorandum No. 2020-0439 Omnibus Interim Guidelines on Prevention, Detection. Isolation Treatment, and Reintegration Strategies for COVID-19. 2020. Available from: https://doh.gov.ph/sites/default/files/health-update/dm2020-0439.pdf

[15] Inter-Agency Task Force for the Management of Emerging Infectious Diseases [IATF]. Omnibus guidelines on the implementation of community quarantine in the Philippines, Amendments May 6, 2021. 2021. Available from: https://doh.gov.ph/sites/default/files/health-update/20210506-OMNIBUS-RRD.pdf

[16] Chang SL, Harding N, Zachreson C, Cliff OM, Prokopenko M. Modelling transmission and control of the COVID-19 pandemic in Australia. Nature communications. 2020 Nov 11;11(1):1-3.

[17] Galanis G, Hanieh A. Incorporating social determinants of health into modelling of COVID-19 and other infectious diseases: A baseline socio-economic compartmental model. Social science & medicine. 2021 Apr 1;274:113794

[18] Anderson SC, Edwards AM, Yerlanov M, Mulberry N, Stockdale JE, Iyaniwura SA, Falcao RC, Otterstatter MC, Irvine MA, Janjua NZ, Coombs D. Quantifying the impact of COVID-19 control measures using a Bayesian model of physical distancing. PLoS computational biology. 2020 Dec 3;16(12):e1008274.

[19] Gallo LG, Oliveira AF, Abrahão AA, Sandoval LA, Martins YR, Almirón M, Dos Santos FS, Araújo WN, de Oliveira MR, Peixoto HM. Ten epidemiological parameters of COVID-19: use of rapid literature review to inform predictive models during the pandemic. Frontiers in Public Health. 2020 Dec 1;8:830.

[20] Hu Z, Ge Q, Li S, Boerwincle E, Jin L, Xiong M. Forecasting and evaluating intervention of Covid-19 in the World. arXiv preprint arXiv:2003.09800. 2020 Mar 22.

[21] Arcede, J.P., Caga-anan, R.L., Mammeri, Y., Namoco, R.A., Gonzales, I.C.A. and Lachica, Z.P., Mata, M.A.E. A modeling strategy for novel pandemics using monitoring data: the case of early COVID-19 pandemic in Northern Mindanao, Philippines. SciEnggJ. 2022 15 (1) 035-046

[22] Galea S, Merchant RM, Lurie N. The mental health consequences of COVID-19 and physical distancing: the need for prevention and early intervention. JAMA internal medicine. 2020 Jun 1;180(6):817-8.

[23] Arcede JP, Caga-Anan RL, Mentuda CQ, Mammeri Y. Accounting for symptomatic and asymptomatic in a SEIR-type model of COVID-19. Mathematical Modelling of Natural Phenomena. 2020;15:34.

[24] Buhat CA, Torres MC, Olave YH, Gavina MK, Felix EF, Gamilla GB, Verano KV, Babierra AL, Rabajante JF. A mathematical model of COVID-19 transmission between frontliners and the general public. Network Modeling Analysis in Health Informatics and Bioinformatics. 2021 Dec;10(1):1-2.

[25] Verma V, Vishwakarma RK, Verma A, Nath DC, Khan HT. Time-to-Death approach in revealing Chronicity and Severity of COVID-19 across the World. PloS one. 2020 May 12;15(5):e0233074.

[26] Verity R, Okell LC, Dorigatti I, Winskill P, Whittaker C, Imai N, Cuomo-Dannenburg G, Thompson H, Walker PG, Fu H, Dighe A. S.[van Elsland], E. Volz, H. Wang, Y. Wang, X. Xi, CA Donnelly, AC Ghani, NM Ferguson, Estimates of the severity of coronavirus disease. 2019:30243-7.

[27] Adam D. MODELLING THE PANDEMIC The simulations driving the world's response to COVID-19. Nature. 2020;580(7803):316-8.

[28] Logrosa G, Mata MA, Lachica ZP, Estana LM, Hassall M. Integrating risk assessment and decision-making methods in Analyzing the dynamics of COVID-19 epidemics in Davao City, Mindanao Island, Philippines. Risk Analysis. 2022 Jan;42(1):105-25.

[29] Van den Driessche P, Watmough J. Reproduction numbers and sub-threshold endemic equilibria for compartmental models of disease transmission. Mathematical biosciences. 2002 Nov 1;180(1-2):29-48.

[30] Zhan J, Liu QS, Sun Z, Zhou Q, Hu L, Qu G, Zhang J, Zhao B, Jiang G. Environmental impacts on the transmission and evolution of COVID-19 combing the knowledge of pathogenic respiratory coronaviruses. Environmental Pollution. 2020 Sep 9:115621.

[31] Hassen HB, Elaoud A, Salah NB, Masmoudi A. A SIR-Poisson Model for COVID-19: Evolution and Transmission Inference in the Maghreb Central Regions. Arabian Journal for Science and Engineering. 2021 Jan;46(1):93-102.

[32] Tang B, Wang X, Li Q, Bragazzi NL, Tang S, Xiao Y, Wu J. Estimation of the transmission risk of the 2019-nCoV and its implication for public health interventions. Journal of Clinical Medicine. 2020 Feb 7;9(2):462.

[33] Philippine Statistics Authority. Highlights of the Region XI (Davao Region) population 2020 Census of population and housing (2020 CPH). 2021 August 23. Available from https://psa.gov.ph/population-and-housing/node/165015

[34] United Nations, Department of Economic and Social Affairs, Population Division (2019). World Population Prospects 2019, Online Edition. Rev. 1. 2019. Available at https://population.un.org/wpp/Download/St

[35] Department of Health. COVID-19 tracker Philippines. 2022 Available at https://doh.gov.ph/covid19tracker

[36] Anonuevo LE, Lato JIE, Amistas DA, Lachica ZPT, Arcede JP, Almocera AES, Mata MAE, de los Reyes V AA. Codes for estimating the baseline epidemiological values of Davao City, Philippines. model-SEIR-COVID-19-Baseline. Available at https://github.com/jelat0/model-SEIR-COVID-19-Baseline/tree/main/Davao%20Baseline%20Paper%20Code

[37] Marino S, Hogue IB, Ray CJ, Kirschner DE. A methodology for performing global uncertainty and sensitivity analysis in systems biology. Journal of theoretical biology. 2008 Sep 7;254(1):178-96.

[38] Massey, S. C. Model Sensitivity Analysis. GitHub.com. 2019 Available from https://github.com/scmassey/model-sensitivity-analysis

[39] Chowell G. Fitting dynamic models to epidemic outbreaks with quantified uncertainty: A primer for parameter uncertainty, identifiability, and forecasts. Infectious Disease Modelling. 2017 Aug 1;2(3):379-98.

[40] Banks HT, Hu S, Thompson WC. Modeling and inverse problems in the presence of uncertainty. CRC Press; 2014 Apr 1.

[41] MATLAB. Version: 9.9.0.1592791 (R2020b) Update 5. Natick, Massachusetts: The MathWorks Inc.; 2020.

[42] Wadhera RK, Wadhera P, Gaba P, Figueroa JF, Maddox KE, Yeh RW, Shen C. Variation in COVID-19 hospitalizations and deaths across New York City boroughs. Jama. 2020 Jun 2;323(21):2192-5.

[43] Katris C. A time series-based statistical approach for outbreak spread forecasting: Application of COVID-19 in Greece. Expert Systems with Applications. 2021 Mar 15;166:114077.

[44] Cori A, Ferguson NM, Fraser C, Cauchemez S. A new framework and software to estimate time-varying reproduction numbers during epidemics. American journal of epidemiology. 2013 Nov 1;178(9):1505-12.

[45] Leung T, Davis SA. Rabies vaccination targets for stray dog populations. Frontiers in veterinary science. 2017 Apr 13;4:52.

[46] Alshammari TM, Altebainawi AF, Alenzi KA. Importance of early precautionary actions in avoiding the spread of COVID-19: Saudi Arabia as an Example. Saudi Pharmaceutical Journal. 2020 Jul 1;28(7):898-902.

[47] Madabhavi I, Sarkar M, Kadakol N. COVID-19: a review. Monaldi Archives for Chest Disease. 2020 May 14;90(2).

[48] Van den Driessche P. Reproduction numbers of infectious disease models. Infectious Disease Modelling. 2017 Aug 1;2(3):288-303.

[49] Shadmi E, Chen Y, Dourado I, Faran-Perach I, Furler J, Hangoma P, Hanvoravongchai P, Obando C, Petrosyan V, Rao KD, Ruano AL. Health equity and COVID-19: global perspectives. International journal for equity in health. 2020 Dec;19(1):1-6.

[50] Gaynor TS, Wilson ME. Social vulnerability and equity: The disproportionate impact of COVID-19. Public administration review. 2020 Sep;80(5):832-8.

[51] Samui P, Mondal J, Khajanchi S. A mathematical model for COVID-19 transmission dynamics with a case study of India. Chaos, Solitons & Fractals. 2020 Nov 1;140:110173.

[52] Wu P, Hao X, Lau EH, Wong JY, Leung KS, Wu JT, Cowling BJ, Leung GM. Real-time tentative assessment of the epidemiological characteristics of novel coronavirus infections in Wuhan, China, as at 22 January 2020. Eurosurveillance. 2020 Jan 23;25(3):2000044.

[53] Bo Y, Guo C, Lin C, Zeng Y, Li HB, Zhang Y, Hossain MS, Chan JW, Yeung DW, Kwok KO, Wong SY. Effectiveness of non-pharmaceutical interventions on COVID-19 transmission in 190 countries from 23 January to 13 April 2020. International Journal of Infectious Diseases. 2021 Jan 1;102:247-53.

[54] Aleta A, Martin-Corral D, Pastore y Piontti A, Ajelli M, Litvinova M, Chinazzi M, Dean NE, Halloran ME, Longini Jr IM, Merler S, Pentland A. Modelling the impact of testing, contact tracing and household quarantine on second waves of COVID-19. Nature Human Behaviour. 2020 Sep;4(9):964-71.

[55] Avancena G, reporter. Hospital in Davao City already at full capacity [television broadcast]. Davao City: ONE News; 2021 September 15

[56] Dubas-Jakobczyk K, Sowada C, Domagała A, Wieckowska B. Building hospital capacity planning mechanisms in Poland: The impact of 2016/2017 regulatory changes. The International Journal of Health Planning and Management. 2018 Apr;33(2):e403-15.

[57] Kerr CC, Stuart RM, Mistry D, Abeysuriya RG, Rosenfeld K, Hart GR, Núñez RC, Cohen JA, Selvaraj P, Hagedorn B, George L. Covasim: an agent-based model of COVID-19 dynamics and interventions. PLOS Computational Biology. 2021 Jul 26;17(7):e1009149.

[58] Atkins JL, Masoli JA, Delgado J, Pilling LC, Kuo CL, Kuchel GA, Melzer D. Preexisting comorbidities predicting COVID-19 and mortality in the UK biobank community cohort. The Journals of Gerontology: Series A. 2020 Nov;75(11):2224-30. https://doi.org/10.1093/gerona/glaa183

[59] Wilder B, Charpignon M, Killian JA, Ou HC, Mate A, Jabbari S, Perrault A, Desai AN, Tambe M, Majumder MS. Modeling between-population variation in COVID-19 dynamics in Hubei, Lombardy, and New York City. Proceedings of the National Academy of Sciences. 2020 Oct 13;117(41):25904-10.

[60] Nishiura H, Jung SM, Linton NM, Kinoshita R, Yang Y, Hayashi K, Kobayashi T, Yuan B, Akhmetzhanov AR. The extent of transmission of novel coronavirus in Wuhan, China, 2020. Journal of clinical medicine. 2020 Jan 24;9(2):330.
