# Supplementary material for: Transmission dynamics and baseline epidemiological parameter estimates of Coronavirus disease 2019 pre-vaccination: Davao City, Philippines
Source: PLoS One. 2023 Apr 7;18(4):e0283068. doi: 10.1371/journal.pone.0283068 (PMC10081793; doi:10.1371/journal.pone.0283068)
Supplement: Attachment — Submitted filename: PONE-D-22-14208.docx [file pone.0283068.s002.docx]

**PONE-D-22-14208**

*1. Is the manuscript technically sound, and do the data support the conclusions?

The manuscript must describe a technically sound piece of scientific research with data that supports the conclusions. Experiments must have been conducted rigorously, with appropriate controls, replication, and sample sizes. The conclusions must be drawn appropriately based on the data presented.*

Partly. The methods employed are standard in disease modelling. The model formulation and parameter definitions are inconsistent in several instances, which could be important for the analysis and the interpretation of the analysis. The specifics are detailed in point number 1 in the below reviewer report.

As the data has not been made available and is not publicly available, the study does not appear to be reproduceable.

The modelling results do support the conclusion; meanwhile, this linkage could be strengthened and the conclusions expanded. The conclusions do not explicitly reference the modelling analysis, could be an area of improvement. Point number 3 in the reviewer report below provides further details.

**2. Has the statistical analysis been performed appropriately and rigorously?*

Yes, the statistical analysis performed in the manuscript (1. LHS/PRCC and 2. Uncertainty quantification via assuming Poisson error structure in the best fit incidence and refitting to each realization for the downstream analysis) are performed appropriately and rigorously.

**3. Have the authors made all data underlying the findings in their manuscript fully available?

The* [*PLOS Data policy*](http://www.plosone.org/static/policies.action#sharing) *requires authors to make all data underlying the findings described in their manuscript fully available without restriction, with rare exception (please refer to the Data Availability Statement in the manuscript PDF file). The data should be provided as part of the manuscript or its supporting information, or deposited to a public repository. For example, in addition to summary statistics, the data points behind means, medians and variance measures should be available. If there are restrictions on publicly sharing data—e.g. participant privacy or use of data from a third party—those must be specified.*

No, information about the data acquisition appears in the manuscript on L149 – 152. In my experience, it is standard for such linelisted epidemiological data (i.e., individual confirmed case data) to be private and not able to share publicly. While the linelist may be private, it might be possible to share the time series extracted from the linelist.

**4. Is the manuscript presented in an intelligible fashion and written in standard English?*

*PLOS ONE does not copyedit accepted manuscripts, so the language in submitted articles must be clear, correct, and unambiguous. Any typographical or grammatical errors should be corrected at revision, so please note any specific errors here.*

The manuscript is overall presented in an intelligible fashion. The authors might consider streamlining the main text and placing non-critical material in an appendix. The English writing and scientific writing could be improved (point number 4 in the below reviewer report). Given the number of typos and grammatical errors, the authors might consider thorough proofreading and editing for the journal readership. Additionally point number 7 in the below reviewer report notes several instances of potential consideration.

**5. Review Comments to the Author

Please use the space provided to explain your answers to the questions above. You may also include additional comments for the author, including concerns about dual publication, research ethics, or publication ethics.*

Additional comments have been provided in the Miscellaneous section in the below reviewer report.

**PONE-D-22-14208**

The manuscript proposes a modified SEIR-type disease transmission model, formulated as a deterministic system of ordinary differential equations, which accounts for several public health measures aimed at mitigating the transmission of SARS-CoV-2. The model was fitted to four phases of interventions in Davao City, Philippines (so called community quarantines), prior to the rollout of vaccination to the population. The model fitting yielded parameter estimates according to each phase of public health interventions. A sensitivity analysis using the Latin hypercube sampling / partial rank correlation coefficient (LHS/PRCC) methodology was performed. Elements of retrospective assessment of the model fit according to different phases of public health measures in Davao City were included. Additionally, several recommendations in terms of public health policy to mitigate transmission of SARS-CoV-2 were provided based on the analysis. The manuscript confronts a relevant scientific problem of the (retrospective) assessment or evaluation of different public health interventions aimed at mitigating transmission of SARS-CoV-2. The topic of intervention assessment in the context of SARS-CoV-2 and other respiratory pathogens is of importance, given the burden of disease and the general need for quantitative evidence to inform policy- and decision- making. I have concerns regarding the model formulation and its novelty. Likewise, the manuscript would benefit from thorough editing for scientific writing and English. Lastly, the linkage between the modelling analysis conducted and public health policy recommendation could be strengthened and, ideally, expanded. Overall, the manuscript would benefit from a revision to address and clarify these points.

**Major points**

Regarding the model (1) formulation and its novelty:

1. The model formulation:
   a. Several parameter definitions are inconsistent with the model formulation. This may be important for both the analysis and the interpretation of the analysis.

- The mean residence time in the E compartment, determined by all relevant outflows, is $\left( \psi\left( 1-r \right)+\delta_{H}rq+\delta_{M}r\left( 1-q \right)+\delta\right)^{-1}$ days. Meanwhile, each removal process has an associated (and different) length of stay in E listed in the description – in contradiction with the model specification. As an example, the quarantine period is written and interpreted in the manuscript as $\Psi^{-1}$ days, the latency periods as $\delta_{M}^{-1}$ and $\delta_{H}^{-1}$ days. However, the mean residence time in E is determined as $\left( \psi\left( 1-r \right)+\delta_{H}rq+\delta_{M}r\left( 1-q \right)+\delta\right)^{-1}$ and will be the same for all individuals entering E, regardless of the compartment to which an individual is transferred to. The same issue is present in the $I_{H}$ and $I_{M}$ compartments, which may also be important, as the dwell time distributions in those compartments determine the distribution of the infectious period. The mean residence time in $I_{H}$ (mean infectious period) is $\left( \mu_{H} +\delta+\gamma_{H} \right)^{-1}$ days. The mean residence time in $I_{M}$ (mean infectious period) is $\left( \phi_{m} m +\gamma\left( 1-m \right)+\delta\right)^{-1}$ days. It is possible to resolve this issue where the residence times are determined as intended; however, it appears to require either the model structure to be revisited or the model parameterized differently.
  b. The stratification of the infectious population and the assumptions about the infectivity of individuals of different statuses would be good to justify, discuss as limitations, and/or revisit. Specifically,
- In the current model, undetected cases (whether presymptomatic, symptomatic or asymptomatic) do not contribute to the force of infection (FOI) whereas those PCR positive cases ($I_{M}$ and $I_{H}$) do contribute to the FOI. Given IPC measures in the hospital setting, one might expect $I_{H}$ to contribute relatively less to the force of infection, compared to undetected infections in the community. Likewise, those mild or asymptomatic cases which have been diagnosed (PCR positive) – individuals in the $I_{M}$ compartment – one would expect are ordered to isolate following their diagnosis and therefore contribute little to the FOI compared to undetected cases. Is it true that individuals who have tested positive are required to isolate in the jurisdiction considered (Davao city)? Overall, the authors might consider adding a compartment for infectious and undetected cases (not PCR positive).
- Individuals in $I_{M}$ and $I_{H}$ contribute equally to the force of infection, i.e., they are assumed to be equally infectious.
  c. Is any aspect of under-reporting or under-ascertainment being considered? In the context of SARS-CoV-2, a substantial proportion of infected individuals typically remain undetected by the surveillance system; however, the model nor the data fitting process does not appear to account for this. Is this not the case in Davao City? This may be particularly relevant for the mild or asymptomatic cases.

2. It is claimed in the manuscript that the model is unique in the context of existing publications in that there is a linkage between E back to S, modelling the release from quarantine among individuals who were not infected. The quarantine and contact tracing process has been modelled in a number of published SARS-CoV-2 transmission dynamics models (e.g., reference [1], see Section 2.2 and Figure 2).

[1] Tang B, Wang X, Li Q, Bragazzi NL, Tang S, Xiao Y, Wu J. Estimation of the transmission risk of the 2019-nCoV and its implication for public health interventions. Journal of Clinical Medicine. 2020 Feb 7;9(2):462.

3. Regarding the relevance of the analysis for public health policy decision-making:
   a. The linkage between the utility of the modelling analysis to support relevant policy- and decision- making and the claims in introduction could benefit from strengthening. Specifically, clear and actionable control guidance based on the analysis conducted, with a reference to the relevant elements of analysis (Figures, Tables, etc.) would be ideal. Control guidance has been provided to some extent in L395 – 420; but, should reference the modelling analysis and ideally be expanded. A demonstration or illustration as to how the modelling results can be utilized to inform PH policy may also allow for the adoption and application of the framework by external users.
4. The manuscript could be clearer and more concise. The manuscript would benefit from thorough editing for scientific writing and English writing.

**Minor points**

5. Limitations of the analysis do not appear to be present in the manuscript. Limitations of the analysis would be good for the authors to include in the manuscript (typically included in Discussion).
6. L327 – 328 and Table 1: Were these parameter values reported in Table 1 estimated from fitting to $M(t)$ or to $H(t)$ (or both)? It would be good to specify the objective function $f(t)$ and explain adequately the process. If applicable, the authors might consider also including parameter tables for both fitting results, allowing for comparison of estimates using different data streams.
7. Several instances of suspected typos and phrasing/terminology that the authors may consider revising in the manuscript are included below. A few suggestions the authors may consider are included:
   a. L6: The word “practice” may be replaced with “implementation”.
   b. L6 and throughout the text: non-pharmaceutical interventions are generally abbreviated as ‘NPIs’.
   c. L19 – 38: The description of the four phases of control measures could be clearer. The authors may consider defining the phases in the order in which they were implemented chronologically (for example) or include a table with their definitions.
   d. L81 and after: traditionally Materials and methods, Results, Discussion and Conclusion are written in past tense.
   e. L96: Suspected typo. “homogenous” may read “homogeneous”.
   f. L129 – 134: The discussion about the uniqueness of the model would generally not be an element of Methods, but of Introduction. Methods typically includes a concise and streamlined description of the methodology employed.
   g. L134 – 135: The cases mentioned could be considered as presymptomatic, i.e., those individuals who have not yet developed symptoms but eventually will develop symptoms.
   h. L177: Suspected typo. “as priori” may read “a priori”.
   i. L210 – 211: The phrasing statistically solved seems nonstandard. The parameter estimation process from data is generally referred to as estimation. The alternative “Other parameters were either estimated from the data [37] or taken from existing published literature” might be clearer.
   j. L271: The authors may consider revising this line. From context, it appears the authors may instead be referring to a unit increase in the parameter which is related to contact tracing.
   k. L395-396: The transmission rate cannot be observed directly – only inferred or estimated. This may more appropriately read: ‘…the transmission rate in Davao City was estimated to be highest during MGCQ…’, or equivalent.
   l. L403: Suspected typo. “closed contacts” 🡪 “close contacts”.
   m. L395 – 420: When referring to the analysis within, reference the appropriate Table or Figure, so as to support control guidance with the modelling analysis.
   n. L395-396: this can be stated more concisely by referencing the expression for the reproduction number.
   o. The authors might consider to place non-critical elements of the main text and figures in an appendix so as to streamline the main text (e.g., the standard mathematical analysis beginning on L329 could be appendix material).

**Miscellaneous**

8. The quality of Figures 2, 4, 5, 6, 7 and 8 should be increased to publication quality.
9. Figure 3. The time-dependent sensitivity analysis may be interpreted in terms of retrospective intervention assessment and public health policy. This may be an element the authors could potentially elaborate upon.
10. The parameter $\beta$can be interpreted as the transmission probability per contact multiplied by the daily per capita contact rate. This interpretation of the estimated transmission coefficients $\beta$ could enter the modelling analysis and subsequent public health policy recommendation (e.g., entering the Discussion in terms of policies based on the corresponding estimates of $\beta$ in each phase). This could add another dimension to the analysis, given that behavior and thus contact rates change temporally as well as the probability of transmission upon contact.
11. L251 – 252, regarding point number 1 concerning the residence times specified in the model: The inverse of the mean residence times in the $E, I_{M}, I_{H}$ compartments can be observed in the calculation of the reproduction number (the parameters defined as A, B and C).
